# Single-cell RNA sequencing reveals endothelial cell heterogeneity and Sox18-mediated EndMT in abdominal aortic aneurysm

**DOI:** 10.7150/thno.110254

**Published:** 2025-08-30

**Authors:** Xianxian Wu, Xuanyu Liu, Yuanzhi Cheng, Yuhan Zhang, Dou Shi, Yang Shi, Xing Liu, Jianghao Feng, Anxiong Long, Wei Hu, Zhiwei Yang

**Affiliations:** 1 Institute of Laboratory Animal Science, Chinese Academy of Medical Sciences (CAMS) & Comparative Medicine Center, Peking Union Medical College (PUMC), Beijing 100021, China.; 2 National Center of Technology Innovation for animal model, Beijing 100021, China.; 3 State Key Laboratory of Cardiovascular Disease, Center of Laboratory Medicine, Fuwai Hospital, National Center for Cardiovascular Diseases, Chinese Academy of Medical Sciences and Peking Union Medical College, Beijing 100037, China.; 4 Beijing Key Laboratory of Cardiovascular Disease Warning and Diagnosis, Beijing 100037, China.; 5 Department of Clinical Pharmacology, The Second Affiliated Hospital of Anhui Medical University, Hefei, 230601, China.

**Keywords:** Abdominal aortic aneurysm, Endothelial cell heterogeneity, EndMT, Sox18, PI3K/Akt signaling pathway

## Abstract

**Rationale:** Abdominal aortic aneurysm (AAA) is a life-threatening cardiovascular disease lacking clinical predictors and effective pharmacologic therapies. The cellular heterogeneity and molecular changes of different cell types during AAA have been revealed in human and mouse aortas by single-cell RNA sequencing (scRNA-seq) technology. However, the heterogeneity and plasticity of endothelial cells (ECs) in AAA remain poorly characterized.

**Methods:** scRNA-seq was performed on the abdominal aorta from angiotensin II (AngⅡ) and salt-induced AAA mice. Additionally, public scRNA-seq data of human and mouse AAA were analyzed with a focus on ECs. Cellular and animal experiments were conducted to validate EC heterogeneity and to investigate the role of SRY (sex-determining region on the Y chromosome)-box transcription factor 18 (Sox18) in endothelial-to-mesenchymal transition (EndMT) during AAA formation.

**Results:** Unbiased clustering analysis identified 20 clusters encompassing 11 cell types. Four subpopulations of ECs were identified in AngⅡ and salt-induced mouse AAA models: *Cd36*^+^ lipid-handling ECs,* Fn1*^+^ mesenchymal-like ECs, *Lrg1^+^* pleiotropically activated ECs, and *Mmrn1*^+^ lymphatic-like ECs. Similar results were observed in human AAA scRNA-seq data. Endothelial dysfunction and EndMT were detected at single cell solution and validated experimentally. Sox18 was identified as a potential EndMT regulator. Sox18 downregulation was confirmed in both human and mouse aortic aneurysm. *In vitro*, Sox18 siRNA transfection induced EndMT and increased EC permeability via PI3K/Akt signaling pathway. *In vivo*, EC-specific Sox18 overexpression inhibited EndMT and attenuated AAA formation.

**Conclusion:** Our data reveal the heterogeneity and transcriptional signatures of ECs in AAA at single cell solution, and demonstrate the previously unrecognized role of Sox18-mediated EndMT in AAA, providing novel insights and a promising therapeutic target for AAA intervention.

## Introduction

Abdominal aortic aneurysm (AAA) is a life-threatening cardiovascular disease that can progress to catastrophic aortic rupture and sudden death if left untreated. The well-characterized features of AAA include vascular smooth muscle cells (VSMCs) degeneration, inflammatory response and extracellular matrix (ECM) degradation, all of which contribute to vascular remodeling and wall weakening 1. Although endovascular aortic repair (EVAR) is highly effective in preventing rupture for eligible AAAs, significant unmet needs remain. Currently, no effective drug therapies are approved to slow or halt the progression of this disease 2. Thus, a better understanding of the molecular and cellular changes in AAA is essential to identify potential therapeutic targets.

Multiple cell types exist in the aorta, primarily including endothelial cells (ECs), VSMCs, macrophages, neutrophils, and fibroblasts 3. The cellular heterogeneity among different cell types is fundamental for normal aortic function. Recently, the systemic transcriptomic landscape and heterogeneity of numerous individual cells in aortic aneurysms have been characterized in both human and mouse using single-cell RNA sequencing (scRNA-seq) technology. In 2021, Zhao *et al.* identified vascular cell transcriptional signatures in a peri-adventitial elastase-induced mouse AAA model, emphasizing VSMCs and monocyte/macrophage responses 4. That same year, Yang *et al.* conducted scRNA-seq using a CaCl₂-induced mouse AAA model to analyze VSMCs, fibroblasts, and macrophages heterogeneity 5. In 2023, Fasolo *et al.* carried out scRNA-seq analysis using four human AAA specimens and discovered that the upregulation of circRNA cATM in VSMCs contributes to AAA 6. However, these scRNA-seq studies paid limited attention to intimal ECs. Although Weng *et al.* described the phenotype of ECs in Angiotensin II (AngII)-induced AAA formation in *ApoE*^-/-^ mice 7, their study lacked in-depth analysis and experimental validation. Therefore, EC heterogeneity and transcriptomic changes in AAA remain incompletely understood.

The EC monolayer forms a selective barrier between blood and vessel walls, playing crucial roles in molecule transportation and signal transduction. Endothelial dysfunction, characterized by an imbalance between vasodilation and vasoconstriction, represents the initiation of many vascular diseases 8. Moreover, ECs possess remarkable plasticity 9, as evidenced by their ability to undergo transformation into mesenchymal cells, a process termed endothelial-to-mesenchymal transition (EndMT). During EndMT, ECs lose their characteristic phenotype and acquire a mesenchymal cell-like phenotype. EndMT has emerged as a key mechanism in vascular pathophysiology 10, implicated in numerous cardiovascular diseases including cardiac fibrosis 11, pulmonary hypertension 12, cerebral cavernous malformations 13, and atherosclerosis 14. A recent study indicates that endothelial tight junction dysfunction contributes to AAA 15, advancing our understanding of aortic aneurysm pathogenesis. However, the role of endothelial dysfunction and phenotype switching in AAA remains poorly understood.

In this study, we performed scRNA-seq on the abdominal aortas from AAA mice induced by AngII and salt, to map the cellular composition during AAA progression. By focusing on ECs and analyzing published scRNA-seq datasets from human and mouse AAA samples, we characterized the transcriptional signatures and EC heterogeneity in aortic aneurysm, with particular attention to the role of Sox18 in EndMT during AAA development. Our findings provide novel insights into the role of ECs in aortic aneurysm, potentially facilitating the identification of non-invasive therapeutic strategies for AAA.

## Materials and Methods

### Human aorta samples

The human abdominal aortic aneurysm (AAA) samples utilized in this study were generously provided by Prof. Chen's research team. These specimens were previously employed in their investigation of SIRT1's role in AAA pathogenesis 16. As detailed in their prior publication, the AAA samples were collected from patients undergoing open surgical repair, with all cases having been preoperatively diagnosed through ultrasound imaging. For control purposes, non-aneurysmal aortic segments were obtained from the same patient cohort. For this study, we used three available matched pairs of samples (Table S1). All protocols involving human aorta specimens were approved by the Ethical Committee of the Chinese Academy of Medical Sciences and Peking Union Medical College, and were conducted in accordance with the Declaration of Helsinki principles and the International Council for Harmonization Guidelines on Good Clinical Practice.

### Animal studies and ethics statement

5-month-old male C57BL/6J mice were purchased from Beijing Vital River Laboratory Animal Technology Co., Ltd., and were housed in a specific pathogen-free (SPF) facility with *ad libitum* access to food and water under a 12 h/12 h light/dark cycle. Only male mice were used in this study because female mice have a lower incidence of aortic aneurysm 17, consistent with the clinical evidence 18. All animal procedures were conducted in accordance with the Guide for the Care and Use of Laboratory Animals published by the US National Institutes of Health (NIH Publication, 8th edition, 2011) and were approved by the Animal Care and Use Committee at the Institute of Laboratory Animal Science, Chinese Academy of Medical Sciences and Peking Union Medical College (approval No. WXX23001).

### Mouse aortic aneurysm model construction

We established a modified angiotensin II (AngII)-induced AAA model using the following protocol: 5-month-old male mice were anesthetized via inhalation of 2-3% isoflurane and administered a single preoperative subcutaneous injection of buprenorphine (0.2 mg/kg) for analgesia. Subsequently, AngII (1500 ng/kg/min; Sigma-Aldrich, #A9525) was continuously delivered for 28 days using subcutaneously implanted osmotic minipumps (Alzet, model 1004). A high-salt solution (0.9% NaCl plus 0.2% KCl) was provided in drinking water throughout the AngII infusion period. After 28 days of AAA induction, mice were euthanized by cervical dislocation under deep isoflurane anesthesia. Following PBS perfusion, abdominal aortas were collected for scRNA-seq analysis, snap-freezing in liquid nitrogen, or fixation in 4% paraformaldehyde (PFA) for subsequent experiments. Mice administered both AngII and a high-salt solution were classified as the AngII+HS group in this study.

### Construction of adeno-associated virus (AAV) serotype 9 vectors for Sox18 overexpression

To induce exogenous expression of Sox18 *in vivo*, we constructed an AAV9 vector system carrying either the plasmid (AAV9-ICAM2-Sox18-p2A) or the scramble control (AAV9-ICAM2-Control) (ViGene Biosciences, Shandong, China). The AAV9 vectors were produced using a triple-plasmid transfection system in HEK293T cells, which included the AAV9 rep/cap plasmid, the adenoviral helper plasmid, and the transgene-containing plasmid. Following transfection, cells were harvested, and the AAV9 particles were purified through iodixanol gradient ultracentrifugation and subsequent dialysis to remove impurities. The purified AAV9 vectors were stored in PBS containing 5% sucrose at -80 ºC until use. For the control, we used an AAV9 vector under the same promoter as that of the experimental vector, which was produced and purified using the same protocol. The viral titer was determined using quantitative PCR (qPCR). Briefly, viral DNA was extracted from the purified AAV9 particles, and the number of viral genomes (vg) was quantified using primers specific to the transgene. The titer was expressed as vector genomes per milliliter (vg/mL). The final titer of the AAV9-Sox18 and AAV9-Control vectors used in this study was 9.10×10^13^ vg/mL and 7.08×10^13^ vg/mL, respectively.

To further corroborate the relationship between Sox18 and AAA, 5-month-old male mice received a single lateral tail intravenous (IV) injection with 2×10^12^ AAV-Sox18 or AAV-Control particles in a total volume of 200 µL PBS. Two weeks post-injection, the mice were subjected to continuous Ang II infusion along with 0.9% NaCl plus 0.2% KCl supplementation in drinking water for 4 weeks. At the experimental endpoint, Sox18 expression levels were assessed in aortic tissues and aortic intima, as well as in other major organs, including the liver, heart and kidneys. Blood pressure (BP) was measured non-invasively using a tail-cuff system (Coda 6; Kent Scientific Corp., Torrington, CT), and aortic diameters were determined by ultrasound imaging.

### Tissue dissociation and sample preparation

The abdominal aortas from control and AngII+HS-induced mice were dissected, minced, and digested in an enzyme solution containing [450 U/mL collagenase type I (Gibco, cat. # 17100-017), 125 U/mL collagenase type XI (Millipore Sigma, cat. # C7657), 60 U/mL hyaluronidase type I-s (Millipore Sigma, cat. # H3506), and 60 U/mL DNase I (Millipore Sigma, cat. #DN25)] for 30 min at 37 ºC. The resulting cell suspension was filtered through a 40 μm cell strainer and washed twice with PBS, then centrifuged at 500 × g for 5 min. Viable cells were resuspended in DMEM supplemented with 10% FBS, and only samples with >80% cell viability were processed for sequencing.

### Single-cell RNA sequencing (scRNA-seq)

Single-cell capture, cDNA Synthesis, and library preparation for scRNA-seq were performed following the manufacturer's protocols. Briefly, single-cell suspensions (300-600 viable cells/μL, verified by CountStar) were processed using the 10× Genomics Chromium system with the Single Cell 3' Library & Gel Bead Kit v3.1 (PN-1000075) and Chromium B Chip (PN-1000074). Cells were resuspended in PBS containing 0.04% BSA and loaded at ~6,000 cells per channel (targeting 50% capture efficiency). Following Gel Bead-in-Emulsion (GEM) generation and cell lysis, reverse transcription was performed in a Bio-Rad S1000TM Thermal Cycler (53 ºC for 45 min; 85 ºC for 5 min, then held at 4 ºC). Amplified cDNA was subjected to quality control using an Agilent 4200 Bioanalyzer (Capital BioTech). According to the manufacturer's instruction, single-cell RNA-seq libraries were constructed using Single Cell 3' Library and Gel Bead Kit V3.1, and final sequencing was performed on an Illumina NovaSeq 6000 platform (paired-end 150 bp [PE150], with ≥100,000 reads per cell).

### Single-cell RNA-seq data preprocessing

Raw sequencing data were processed using the Cell Ranger 7.0 pipeline with default settings. FASTQ files generated from Illumina sequencing were aligned to the GRCm39 mouse genome assembly. Gene-barcode matrices were constructed for each sample by quantifying unique molecular identifiers (UMIs) and filtering out non-cell-associated barcodes. These data were imported into the Seurat (v4.0.0; R package) for quality control and subsequent analysis. Low-quality cells were filtered out based on a standardized set of three quality criteria: (1) the number of detected transcripts, quantified by UMIs (≥500 UMIs/cell), (2) the number of detected genes (≥ 200 genes/cell), and (3) the percentage of reads mapping to mitochondrial genes (< 20%). Doublets were removed using the DoubletFinder (v2.0.3; R package). The percentage of reads mapping to mitochondrial genes was calculated using the “PercentageFeatureSet” function of the Seurat package. The count matrices were normalized using the “NormalizeData” function. Variable genes were identified using the “FindVariableFeatures” function. Following data integration using the “FindIntegrationAnchors” and “IntegrateData” functions, we applied principal component analysis (PCA) and uniform manifold approximation and projection (UMAP) for dimensionality reduction. Cell clustering was performed using the Louvain algorithm on a shared nearest neighbor graph constructed from PCA results 19. Cluster marker genes were identified by comparing the expression levels between the cluster and the remaining clusters using the Wilcoxon rank-sum test implemented in the “FindMarkers” function in Seurat.

### Identification of differentially expressed genes (DEGs) and pathway enrichment analysis

DEGs between groups were detected using the Wilcoxon rank-sum test implemented in the “FindMarkers” function of Seurat. Genes were considered significant if they met the following criteria: **|**log_2_(fold change)**|** > 0.5, Benjamini-Hochberg adjusted p-value < 0.05, and expression in ≥10% of cells in at least one group. Functional enrichment analysis of the DEGs was performed using Metascape 20, with Gene Ontology Biological Process (GO-BP) and Kyoto Encyclopedia of Genes and Genomes (KEGG) pathways as the primary databases. The significance threshold was set at a false discovery rate (FDR) *q*-value < 0.05 based on the hypergeometric test.

### Regulon analysis

To examine the role of transcriptional regulators in specific cell types and in the clusters of vascular endothelial cells (VECs) during aortic aneurysm progression, we performed single-cell regulatory network inference and clustering (SCENIC) 21. The input matrix consisted of the normalized feature-barcode expression matrix. The workflow comprised three main steps: (1) co-expression network inference using the GENIE3 R package to identify potential transcription factor (TF)-target relationships; (2) motif enrichment analysis with the RcisTarget R package, which evaluates gene-motif associations within genomic regions spanning from 500 bp upstream to 100 bp downstream of the transcription start site (TSS); and (3) regulon activity scoring using the AUCell R package, where the top 5% of genes (aucMaxRank = 5%) were included for activity score calculation. A default binarization threshold was then applied to convert continuous activity scores into binary states, with “0” indicating the TF is inactive (“off”) and “1” indicating it is active (“on”). The average regulon activity scores in the subpopulation were used to identify subpopulation-specific regulons. Cell type- or cluster-specific regulons were determined by comparing activity scores across groups using the Wilcoxon rank-sum test (adjusted *p*-value < 0.05).

### Gene set enrichment analysis

Gene set enrichment analysis (GSEA) was performed using the GSEA software (version 4.0.1), with predefined gene sets from the Molecular Signatures Database (MSigDB v2023.2). Gene ranking was performed using the Signal2Noise algorithm, which calculates the standardized mean difference between groups normalized by standard deviation. The ranked gene list was analyzed using GSEA with a significant threshold of FDR *q*-value < 0.05. The minimum and maximum criteria for selection of gene sets from the collection were 0 and 500 genes, respectively.

### Public human and mouse aortic aneurysm scRNA-seq datasets

Raw scRNA-seq data of human AAA specimens were obtained from the Gene Expression Omnibus (GEO) database under accession number GSE237230 6. Mouse AAA data (AngII-induced: GSE186865 22; CaCl₂-induced: GSE164678 5) were similarly retrieved. All sequencing data were processed using Seurat (v5.0.1) for each sample. Cells with >25% of reads mapping to mitochondrial genes and cells with fewer than 200 or more than 2,500 detected genes were filtered out. Data normalization was performed using the “LogNormalize” method (scale factor = 10,000) via the “NormalizeData” function. Highly variable features were identified using the “FindVariableFeatures” function with the “vst” method. For sample integration, the “FindIntegrationAnchors” and “IntegrateData” functions were employed. The standard workflow for visualization and clustering was followed, using the “RunUMAP”, “FindNeighbors”, and “FindClusters” functions. Endothelial cells (ECs) were identified by canonical markers (*Cdh5*, *Kdr*, *Pecam1* and *Mmrn2*) and isolated for subpopulation analysis. GO and KEGG enrichment analyses were conducted using clusterProfiler (v4.6.2) on DEGs between distinct EC clusters or experimental conditions (AngII vs. PBS and AAA vs. Sham).

### Bulk RNA-seq of mouse aortic aneurysm induced by deoxycorticosterone acetate (DOCA) plus salt

Previously, we performed bulk RNA-seq analysis of aortic tissues from control mice (n = 5) and DOCA plus salt-induced mice (n = 3). The RNA-seq data were deposited in the GEO database with accession number GSE153425. For the current analysis, we examined expression changes of selected genes, including: transcription factors (*Sox4*, *Sox6*, *Sox7*, *Sox9*, *Sox17*,* Sox18*, and *Gata6*), endothelial cell markers (*Pecam1*, *Cdh5*, *Cldn5*, *Vwf*, *Tie1*, and *Cdh13*), and mesenchymal cell markers (*Fn1*, *Fbn1*, *S100a4*, *Col1a1*, *Col1a2*, *Col3a1*, and *Col12a1*).

### Bioinformatic analysis of microarray data of aortic tissues from human AAA samples and mouse AAA samples induced by AngⅡ

Microarray data from aortic tissues of human AAA samples (GSE57691) and from AngII-induced *ApoE*^-/-^ mice (GSE17901) were downloaded from the GEO database. The data were normalized using the standard normalization procedures as described in the original studies 23, 24. We focused on the expression changes of Sox18 and EndMT-related genes.

### Plasma lipids detection

Plasma biochemical parameters, including low-density lipoprotein cholesterol (LDL-C), triglyceride (TG), total cholesterol (TCHO), high-density lipoprotein cholesterol (HDL-C) were measured using a Mindray BS-360 automatic biochemical analyzer with manufacturer-specified reagents (Mindray, China).

### Transmission electron microscope (TEM)

To observe the ultrastructural changes in aortic endothelial cells during aortic aneurysm development, 1 mm³ aortic tissue samples were processed for TEM. Freshly excised tissues were fixed in 2.5% glutaraldehyde (in 0.1 M sodium cacodylate buffer, pH 7.4) for 2 h at 4 ºC, post-fixed in 1% osmium tetroxide for 1 h, and washed with 0.1 M sodium cacodylate buffer. After gradient alcohol dehydration, tissues were embedded in Epon 812 resin. Semi-thin sections (1 μm) were stained with 1% methylene blue for light microscopic examination. Ultra-thin sections were double-stained with uranyl acetate and lead citrate, and then observed under a JEM-1400 electron microscope.

### Western blotting

Total protein was extracted from endothelial cells using RIPA lysis buffer containing 100× protease inhibitor cocktail (Sigma-Aldrich) and 10× PhosSTOP phosphatase inhibitor (Roche, Basel, Switzerland). Protein concentrations were determined using the enhanced BCA Protein Assay Kit (Beyotime). Equal amounts of protein were loaded on SDS-PAGE gels and then transferred to nitrocellulose membranes. Membranes were blocked with 5% (w/v) non-fat dry milk in TBST for 1 h at room temperature to prevent nonspecific binding. After blocking, membranes were incubated with the following primary antibodies overnight at 4 ºC: CD31 (1:1000, ab28364, Abcam), CD31 (1:1000, sc-376764, Santa Cruz), α-SMA (1:1000, ab7817, Abcam), Vimentin (1:1000, #5741, Cell Signaling Technology), and Sox18 (1:1000, #PA5-40640, Invitrogen). After washing, membranes were incubated with horseradish peroxidase (HRP)-conjugated secondary antibodies at room temperature for 1 h. Subsequently, protein bands were visualized using enhanced chemiluminescence (ECL) substrate on a Tanon 5500 Chemiluminescent Imaging System (Tanon, Shanghai, China).

### Aortic CD31^+^ ECs isolation

To analyze gene expression in aortic ECs, CD31^+^ ECs were isolated using a magnetic bead-based positive selection method as previously described 25. Briefly, aortas from control and AngII+HS-treated mice were collected after intracardiac perfusion with ice-cold PBS. The aortas were cut into 1-2 mm segments and digested in an enzyme solution containing 450 U/mL collagenase type I, 125 U/mL collagenase type XI, 60 U/mL DNase I, and 60 U/mL hyaluronidase at 37 ºC for 60 min. The digestion was stopped by adding FACS buffer supplemented with 2 mM EDTA and 2% FBS. The cell suspension was filtered through a 30-μm strainer to remove aggregates, then incubated with CD31 MicroBeads (Miltenyi Biotec) on ice for 15 min. CD31^+^ cells were separated using an LS column (Miltenyi Biotec) equipped with a magnet. Before cell collection, the column was washed three times with FACS buffer to remove unbound cells. The purified ECs were then processed for protein extraction.

### RNA extraction and real-time RT-PCR

Total RNA extraction from tissues and cells was performed using TRIzol reagent (Invitrogen, CA, USA). For isolating intimal RNA from mouse aortas, we rapidly flushed the lumen with TRIzol reagent using an insulin syringe, and then collected the eluate into a 1.5 mL tube for RNA extraction, as previously described 26, 27. RNA concentrations were measured using a NanoDrop spectrophotometer (Thermo Fisher Scientific), and cDNA was synthesized from 1 μg total RNA using the High-Capacity cDNA Reverse Transcription Kit (Applied Biosystems, Foster City, CA, USA). Quantitative real-time PCR (qRT-PCR) was performed using SYBR Green PCR Master Mix (RR820A, TaKaRa) on a StepOnePlus Real-Time PCR System (Applied Biosystems). Relative mRNA expression levels were calculated using the 2^-ΔΔCt^ method, with β-actin serving as the internal control. The primer pairs used in this study are listed in Table S2.

### Cell culture and treatment

Human aortic endothelial cells (HAECs) were purchased from ScienCell Research Laboratories and were cultured in endothelial cell medium (pH 7.4) containing 5% fetal bovine serum (FBS), 1% endothelial cell growth factors, and 1% penicillin/streptomycin. Cells at passages 5-7 were seeded in 6-well or 12-well culture plates (Corning) and then transfected with 100 nM siRNA per well using Lipofectamine™ 3000 (Invitrogen, #L3000008) following the manufacturer's protocol. Sox18 siRNA and negative control siRNA were synthesized by OBiO Technology (Shanghai, China). The Sox18 siRNA sequences were sense, 5'-GCGCGUGCAUCUCCGGCUAGTT-3', and antisense, 3'-CUAGCCGGAGAUGCACGCGCTT-5'. The negative control (NC) sequences were sense, 5'-UUCUCCGAACGUGUCACGUTT-3', and antisense, 3'-ACGUGACACGUUCGGAGAATT-5'. To inhibit PI3K/Akt signaling, cells were treated with either LY294002 (20 μM) or XL147 (2 μM) (both from MedChemExpress, Shanghai, China). Following treatment, cells were harvested for immunofluorescent staining, protein and RNA extraction, and permeability assays.

### Determination of endothelial cell permeability

Endothelial cell permeability was determined using fluorescein isothiocyanate (FITC)-dextran (40 kDa; FD40S; Sigma-Aldrich) according to the previously described methods 27, 28. Briefly, HAECs were seeded on Transwell culture inserts (pore size, 0.4 μm; 3413; Corning) with complete ECM medium and cultured for 24 h, followed by transfection with Sox18 siRNA for 48 h. Subsequently, the medium of the Transwell apical compartment was replaced with 200 μL fresh medium containing 1 mg/mL FITC-dextran, and 600 μL fresh medium was added to the basal compartment. After incubation for 1 h and 2 h, 50 μL aliquots were collected from the basal compartment and transferred to a 96-well plate. The fluorescence intensity of FITC-dextran in this basal medium was measured using a microplate reader (Wallac Victor3 1420 multilabel counter; PerkinElmer).

### Immunofluorescence staining

Immunofluorescence staining was performed on the following samples: (1) paraffin-embedded sections of human AAA and adjacent normal aortic segments, (2) frozen sections of mouse abdominal aorta, and (3) cultured ECs. For paraffin sections, antigen retrieval was conducted in 0.01 M citrate buffer (pH 6.0) after deparaffinization. All samples were fixed in 4% PFA solution for 30 min, permeabilized in 0.1% Triton X-100 (Sigma) in PBS buffer and blocked with 2% goat serum (Sigma) for 1 h at room temperature. Samples were then incubated with primary antibodies overnight at 4 ºC, followed by appropriate fluorescein-labeled secondary antibodies (1:500) for 1 h at room temperature in the dark. After mounting with DAPI-containing fluorescent mounting medium (ZLI-9557, ZSGB-BIO), images were acquired using a Fluoview FV1000 confocal microscope (Olympus, Tokyo, Japan) and analyzed with ImageJ software. We used the following primary antibodies for immunofluorescence staining: CD31 (1:100, ab28364, Abcam), CD31 (1:100, sc-376764, Santa Cruz), α-SMA (1:100, ab7817, Abcam), CD36 (1:100, A14714, ABclonal), FN1 (1:200, 66042-1-Ig, Proteintech), LRG1 (1:100, A7850, ABclonal), MMRN1 (1:100, A6658, ABclonal).

### Histology

Paraffin-embedded mouse abdominal aortas were sectioned into 6-μm slices and used for hematoxylin and eosin (H&E) staining, Masson's trichrome staining (G1346, Solarbio), and elastic fiber staining (G1593, Solarbio) according to standard protocols.

### Statistical analysis

All experimental data were expressed as mean ± SEM and were analyzed using GraphPad Prism software 8.0. Aortic aneurysm incidence was analyzed by Fisher's exact test. Data normality was checked using the Shapiro-Wilk test. For comparisons between two groups, the standard Student's t-test was used for normally distributed data, and Mann-Whitney nonparametric test was used for nonnormally distributed data. For comparisons among more than two groups, one-way or two-way ANOVA with Tukey's multiple comparisons test was used for normally distributed data. Otherwise, Kruskal-Wallis test with Bonferroni correction was applied. A *P* value < 0.05 was considered statistically significant.

## Results

### Ang Ⅱ and salt induce AAA in C57BL/6J mice

AAA was successfully induced by AngII infusion and salt administration (AngII+HS) in C57BL/6J mice. Ultrasound imaging revealed significant increases in both maximal internal diameter and wall thickness of abdominal aortas in AngII+HS-induced mice (Figure S1A-C). Additionally, mice in the AngII+HS group exhibited a significantly higher AAA incidence rate and enlarged external aortic diameters (Figure S1D-F). Meanwhile, both systolic and diastolic blood pressure were obviously elevated in the AngII+HS group (Figure S1G-H). Histopathological analysis demonstrated that AngII+HS induced elastin degradation and collagen deposition (Figure S1I), reproducing the pathological features of human aortic aneurysm.

### Global cellular landscape in mouse AAA induced by AngⅡ and salt

**Figure 1:**
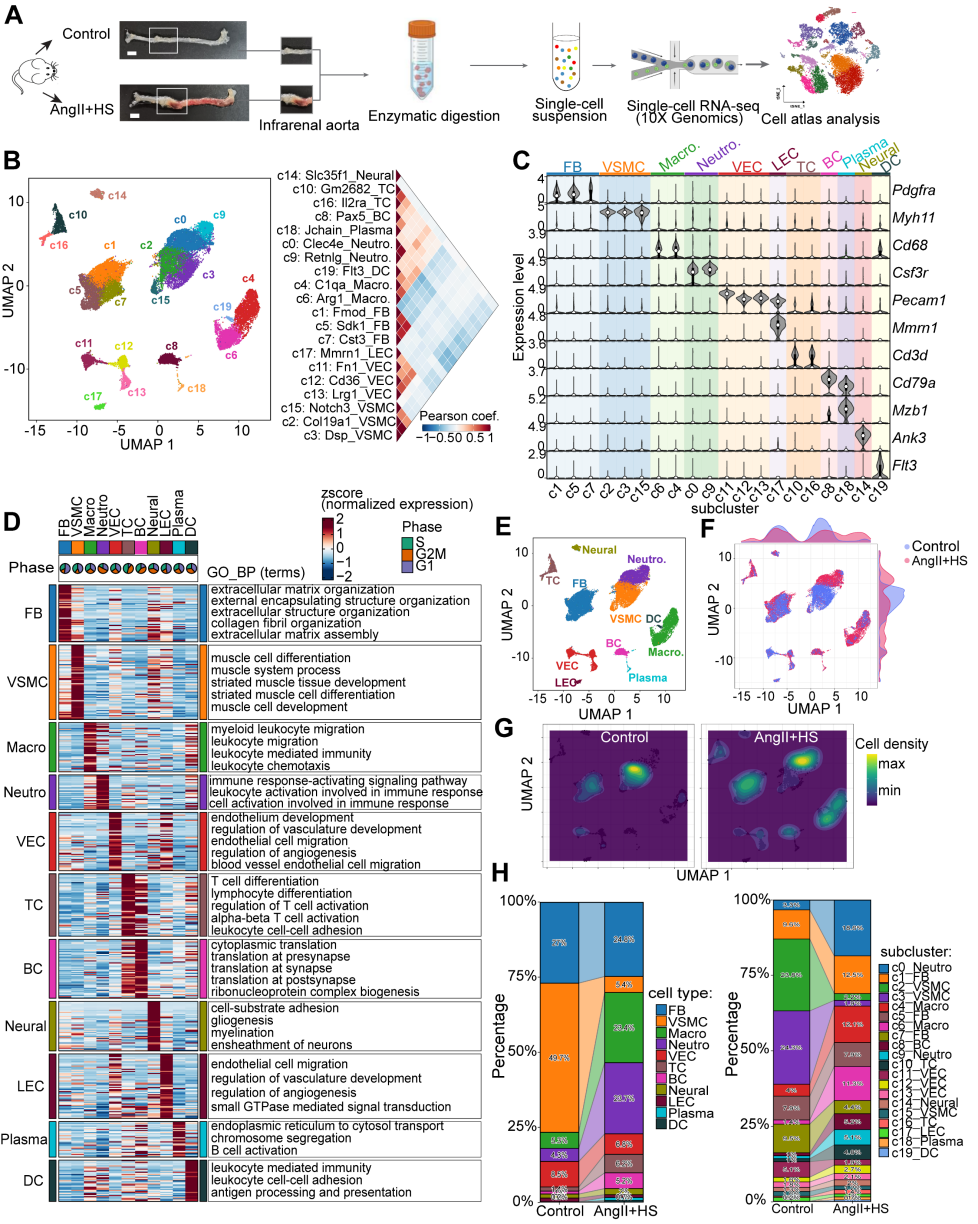
**Single-cell RNA sequencing (scRNA-seq) analysis of cell type composition in mouse aortic tissue from control and aortic aneurysm samples.** (A) Schematic of the scRNA-seq experimental workflow. Abdominal aortas were harvested 28 days after induction with AngⅡ+HS or saline. Aortic tissues from five mice were pooled for each condition for scRNA-seq. Scale bar = 2 mm. (B) Unsupervised clustering of the combined dataset (28,707 cells from all samples) identified 20 cell clusters. The right panel displays Pearson's correlation coefficients between the 20 cellular clusters. (C) Expression levels of canonical marker genes for the identified cell types across clusters. (D) Heatmap depicting expression patterns and functional enrichment of cell type-specific genes. Rows represent z-score-normalized expression values. Representative Gene Ontology Biological Process (GO-BP) terms are listed for each cell type. Pie charts indicate the percentage of cells in each phase of the cell cycle. (E) Uniform manifold approximation and projection (UMAP) plot of the 11 identified cell types. Relative expression of several marker genes in major cell types from all samples. (F) UMAP projection of cells colored by group identity. (G) Kernel density visualization highlighting proportional changes for smooth muscle cells, neutrophils and macrophages in AngⅡ+HS group compared to control group. (H) Stacked bar plots showing the relative proportion of each cell type (left) and cluster (right) per group. FB, fibroblasts; VSMC, vascular smooth muscle cell; Macro, macrophage; Neutro, neutrophil; VEC, vascular endothelial cell; TC, T cell; BC, B cell; Neural, neural cell; LEC, lymphatic endothelial cell; Plasma, plasma cell; DC, dendritic cell.

The abdominal aorta of mice from the control group and AngII+HS-induced group was dissociated for single-cell RNA sequencing (scRNA-seq) analysis (Figure 1A). After stringent quality control, a total of 28,707 qualified cells were retained for further biological analysis (Figure S2). Following data integration, normalization and unbiased clustering, a total of 20 clusters were initially obtained (Figure 1B). Based on the expression of canonical markers for aortic cell types and their transcriptional similarity, the clusters were annotated as 11 major cell types (Figure 1B-C). The 11 major cell types included 3 clusters of fibroblasts (FB, marked by the expression of *Dcn*, *Lum*, *Col1a1*, *Col3a1*), 3 clusters of vascular smooth muscle cells (VSMC, marked by the expression of *Myh11*, *Tagln*, and *Acta2*), 2 clusters of macrophages (Macro, marked by the expression of* Cd68*, *Cdca3*, and *C1qb*), 2 clusters of neutrophils (Neutro, marked by *Ccr1*, *S100a8*, and* S100a9*), 3 clusters of vascular endothelial cells (VEC, marked by the expression of *Cdh5*, *Pecam1*, and *Fabp4*), lymphatic endothelial cells (LEC, marked by the expression of *Cdh5*, *Prox1*, and *Mmrn1*), 2 clusters of T cells (TC, marked by the expression of *Cd3d*, *Cd3g*, and *Cd28*), B cells (BC, marked by the expression of *Cd79a*, *Cd19*, and *Cd79b*), plasma cells (marked by the expression of *Mzb1*, *Cd79a*, and *Tnfrsf13b*), dendritic cells (DCs, marked by the expression of *Cd209a*, *Ccr7*, and *Ifitm1*), and neural cells (marked by the expression of *Ank3*, *Kcn1*, and *Mpz*) (Figure 1B-C, Table S3). Clusters annotated as the same cell type exhibited concordant expression patterns of canonical marker genes (Figure 1C). Each cell type had distinct gene signatures, as shown in Figure 1D. Functional enrichment analysis of cell-type signatures further confirmed their identities (Figure 1D, Table S4). UMAP visualization revealed changes in the relative proportion of certain cell types in the aortas of mice induced by AngII+HS (Figure 1E-G). Further quantitative analysis of cellular composition revealed a reduction in the proportion of VSMCs, alongside increased proportions of macrophages and neutrophils, in the AngII+HS group compared to the control group (Figure 1H), consistent with vascular degeneration and inflammatory progression features in AAA 29, 30.

### The heterogeneity of ECs in the development of aortic aneurysm

**Figure 2:**
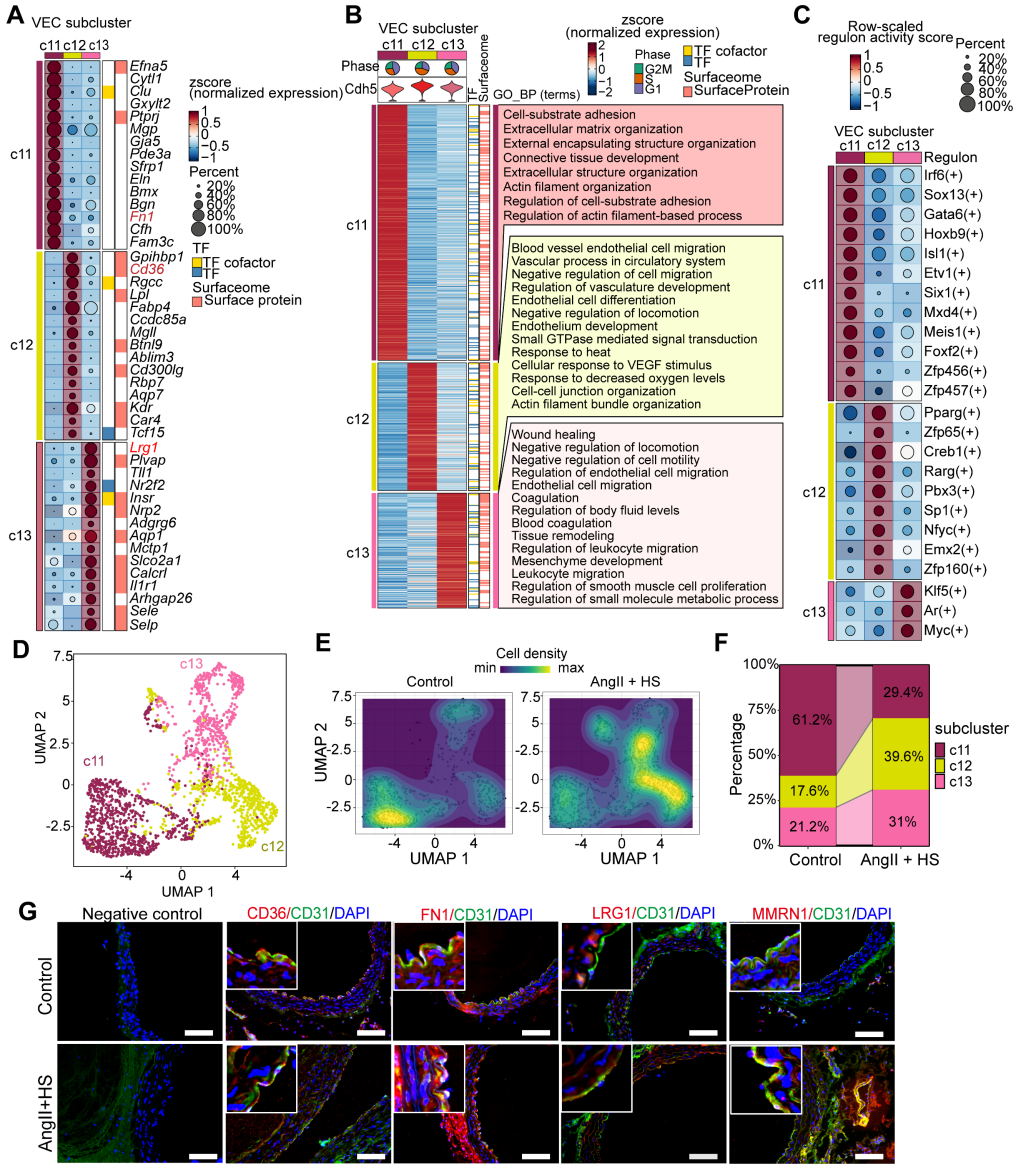
** Endothelial cell (EC) heterogeneity in mouse abdominal aortic aneurysm (AAA).** (A) Dot plot showing expression of top marker genes for each VEC cluster. Dot size indicates the percentage of cells expressing each gene, and dot color indicates expression level. (B) Heatmap showing the expression of signature genes across VEC clusters, with representative enriched GO-BP terms. (C) Dot plot highlighting regulons with cluster-specific activity. The dot color indicates the average regulon activity, as inferred using SCENIC. (D) UMAP visualization of the three VEC clusters. (E) Visualization of cellular density reveals proportion changes of the three VEC populations. (F) Stacked bar plot showing relative proportions of the three VEC clusters. (G) Immunofluorescence staining indicating the presence of Cd36^+^ (c12), Fn1^+^ (c11), Lrg1^+^ (c13) VECs, and Mmrn1^+^ LECs (c17) in aortas of control mice and AngⅡ+HS-treated mice. Scale bar = 100 μm.

Endothelial dysfunction has been identified as a precursor to AAA formation 8. In this study, we characterized distinct EC subpopulations and their functional properties in AAA to provide both structural insights and potential therapeutic targets for AAA. ScRNA-seq analysis revealed four EC subpopulations (c11, c12, c13, and c17) (Figure 1B-D), with c17 identified as lymphatic ECs (LECs) and c11, c12, and c13 as vascular ECs (VECs). Although c17 and VECs shared some transcriptional similarities, c17 exhibited significantly higher expression of *Mmrn1*, a carrier protein involved in platelet aggregation regulation, along with *Prox1*, the definitive transcription factor establishing lymphatic endothelial identity (Figure 1C, Table S3). Notably, c11, c12 and c13 were VECs with distinct expression profiles and specialized functional signatures (Figure 2A-B, Table S5). c11 showed a fibrotic signature, marked by high expression of extracellular matrix genes (such as *Fn1*, *Bgn*,* Eln*, and *Mgp*), suggesting active collagen fiber assembly. c12 displayed a lipid-metabolic phenotype, characterized by elevated *Cd36* and *Gpihbp1* expression, indicative of enhanced lipid processing. c13 demonstrated a pronounced inflammatory activation profile, with upregulation of endothelial adhesion molecules (*Lrg1*, *Selp*, *Sele*, and *Vcam1*) alongside immune-related genes (*IL1r1*, *H2-Q7*, and *Ly6c1*), implicating its role in vascular inflammation and immune response (Figure 2A, Table S5). Functional enrichment analysis further confirmed these specialized roles, with c11 associated with matrix organization and tissue development, c12 with endothelium development and junction organization, and c13 with pathways related to cell migration, coagulation, mesenchyme development, and leukocyte recruitment (Figure 2B). Based on these findings, we designated these subpopulations as *Cd36*^+^ lipid-handling ECs (c12), *Fn1*^+^ mesenchymal-like ECs (c11), *Lrg1*^+^ activated ECs (c13), and *Mmrn1*^+^ lymphatic ECs (c17), each contributing uniquely to AAA pathogenesis through their distinct molecular and functional characteristics.

Furthermore, we performed SCENIC analysis to identify subpopulation-specific transcriptional regulons. As shown in Figure 2C, c11 subpopulation exclusively expressed *Etv1* and *Six1* as its characteristic transcription factors (TFs), while* Zfp65* was specifically enriched in the c12 subpopulation. Notably, two lipid metabolism-associated TFs, *Pparg* and *Creb1*, exhibited their highest regulatory activity in c12. In contrast, *Klf5* and *Myc* showed both greater specificity and enhanced activity in the c13 subpopulation.

The three VEC subpopulations were clearly distinguishable in the UMAP visualization (Figure 2D). Comparative analysis of cellular distribution revealed significant shifts in subpopulation abundance between AngII+HS-treated and control aortas (Figure 2E). Specifically, AngII+HS treatment led to a reduction in the c11 subpopulation accompanied by an expansion of c12 and c13 subpopulations (Figure 2F). Subsequently, the four EC subclusters were experimentally validated in abdominal aorta of AAA mice induced by AngII+HS (Figure 2G), as well as in human AAA samples (Figure S3) through immunofluorescence staining.

To validate our EC findings, we analyzed a published scRNA-seq dataset of AngII-induced AAA in *ApoE*^-/-^ mice 22. ECs were identified using established markers (*Cdh5*, *Kdr*, *Pecam1*, and *Mmrn2*). This independent analysis confirmed our initial observations, with ECs similarly clustering into four distinct subpopulations exhibiting unique transcriptional profiles (Figure S4A-B, Table S6). EC1 specifically expressed lipid-handling genes, including *Cd36*, *Fabp4*, *Gpihbp1*, and *Lpl*; EC2 specifically expressed *Smoc1*, a regulator of osteoblast differentiation; EC3 specifically expressed *Mmrn1, Reln,* and* Pdpn*; EC4 showed high levels of activation marker *Lrg1* and inflammation-associated genes (*Ccdc3*,* Il6st*), indicating an activated EC subpopulation with pro-inflammatory state (Figure S4B). GO enrichment analysis (Table S7) showed that, EC1 was enriched for vasculature development, such as regulation of angiogenesis, endothelium development (Figure S4C). EC2 exhibited enrichment in extracellular matrix organization, connective tissue and muscle development and cell-substrate adhesion, possessing the characteristics of both endothelial and mesenchymal cells (Figure S4D). EC3 showed enrichment in leukocyte migration, blood coagulation and platelet aggregation, indicating a role in hemostasis regulation (Figure S4E). EC4 showed enrichment in tissue migration, epithelial migration and leukocyte migration (Figure S4F). Cross-dataset comparison showed conserved identities: EC1 (corresponding to our c12 cluster, *Cd36*^+^ lipid-handling ECs), EC2 (c11, *Fn1*^+^ mesenchymal-like ECs), EC3 (c17, *Mmrn1*^+^ lymphatic-like ECs), and EC4 (c13, *Lrg1^+^* pleiotropically activated ECs), underscoring the biological relevance of these EC subpopulations in AAA pathogenesis.

To bridge our murine scRNA-seq findings to human AAA pathology, we analyzed public scRNA-seq data from AAA patients with a focus on ECs 6. Strikingly, we identified four conserved EC subtypes in human samples (Figure S5A), each exhibiting unique molecular signatures (Figure S5B, Table S8). EC1 exhibited an activated phenotype, marked by elevated expression of antigen presentation molecules (*CD74*, *HLA-DRA*) and significant enrichment in immune activation pathways including cytokine signaling and leukocyte migration (Figure S5C, Table S9). EC2 demonstrated a fibrotic signature characterized by co-expression of extracellular matrix genes (*MGP*, *S100A4*, *FN1*, *COL8A1*) and coagulation factors (*F5*), with functional enrichment in connective tissue development, extracellular matrix organization and blood coagulation, implying the mesenchymal-like and pro-coagulation features of this subpopulation (Figure S5D). EC3 maintained typical endothelial functions, showing high expression of junctional proteins (*JUP* 31, *EDIL3* 32) and enrichment in cell-substrate adhesion negative regulation of cell migration pathways, reflecting its normal EC functions in maintaining the expression of cell junction molecules. Most intriguingly, we identified a fourth subtype (EC4) with prominent ribosome biogenesis activity (Figure S5F), likely reflecting heightened metabolic requirements 33 and potential mesenchymal transition characteristics 34. While this analysis confirmed the presence of conserved EC subtypes (lipid-handling, mesenchymal-like, activated) in human AAA, it revealed an absence of canonical LECs but identified a distinct EC subcluster with prominent ribosome biogenesis activity.

### ECs exhibits different transcriptional phenotypes and molecular characteristics in the context of aortic aneurysm

**Figure 3:**
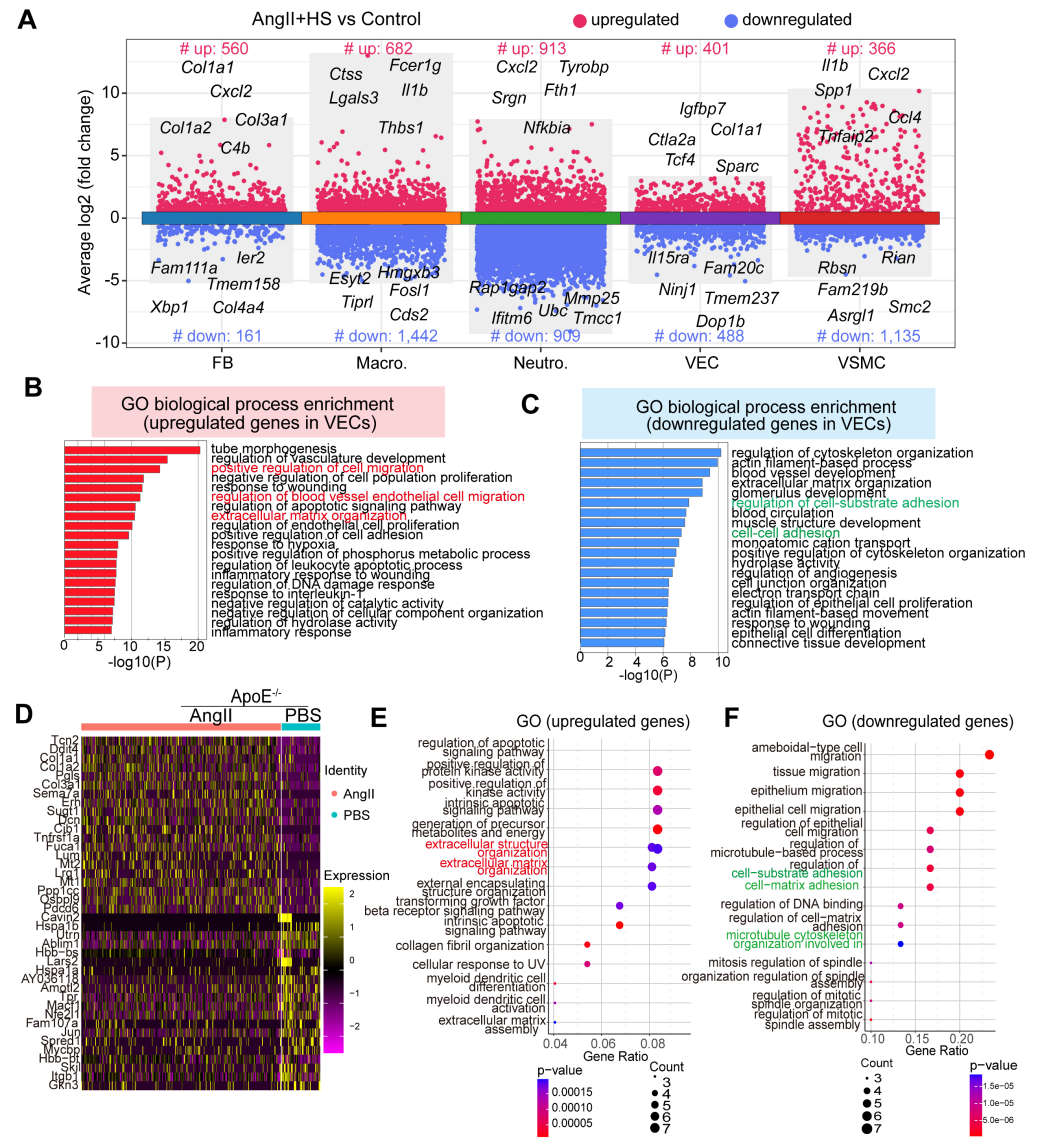
** Transcriptomic dysregulation in vascular ECs following AngⅡ+HS challenge.** (A) Volcano plot presenting differentially expressed genes (DEGs) in major cell types of AngⅡ+HS group compared to the control group. (B) GO-BP enrichment analysis of the upregulated genes in VECs of AngⅡ+HS group vs. control group. (C) GO enrichment analysis of the downregulated genes in VECs of AngⅡ+HS group vs. control group. (D) Heatmap of DEGs in aortic ECs from AngⅡ- or PBS-treated *ApoE*^-/-^ mice revealed by scRNA-seq analysis. LogFC ≥ 0.25. (E) GO enrichment analysis of the upregulated DEGs in AngⅡ- vs. PBS-treated *ApoE*^-/-^ mice. (F) GO enrichment analysis of the downregulated DEGs in AngⅡ- vs. PBS-treated *ApoE*^-/-^ mice.

To delineate the molecular changes of ECs in the development of AAA, we conducted differential gene expression analysis. A total of 401 significantly upregulated and 488 significantly downregulated genes were identified when comparing ECs of the AngII+HS group with those of the control group (Figure 3A). GO enrichment analysis revealed that the upregulated genes were mainly enriched in pathways associated with extracellular matrix organization, positive regulation of cell migration and inflammation (Figure 3B and Table S10). In contrast, the downregulated genes were mainly associated with cell-cell adhesion, cell-substrate adhesion and cytoskeleton organization regulation (Figure 3C and Table S11). Gene set enrichment analysis (GSEA) revealed significant activation of epithelial-to-mesenchymal transition, extracellular matrix organization and immune response in ECs of AngII+HS-treated mice (Figure S6 and Table S12), suggesting EC activation.

Moreover, analysis of a published scRNA-seq dataset identified a set of differentially expressed genes in ECs from abdominal aorta samples of PBS- and AngII-treated *ApoE*^-/-^ mice 22, including cytoskeleton-related genes (e.g.,* Macf1, Utrn*) and ECM genes (e.g.*, Lum, Dcn, Col1a1,* and* Col3a1*) (Figure 3D). GO enrichment analysis showed that the upregulated genes were mainly enriched in extracellular matrix organization (Figure 3E), whereas the downregulated genes were associated with the regulation of cell-matrix adhesion and microtubule cytoskeleton organization (Figure 3F). In the CaCl_2_-induced mouse model of AAA, analysis of the published scRNA-seq datasets 5 revealed similar transcriptional alterations in ECs between the AAA group and the sham group. Specifically, the upregulated genes were predominantly enriched in pathways related to extracellular matrix organization and muscle tissue development. Conversely, the downregulated genes were mainly associated with cell-cell and cell-substrate adhesion pathways (Figure S7A-B).

Collectively, these data demonstrate that AAA progression involves profound transcriptional reprogramming of ECs, characterized by progressive loss of canonical endothelial features (such as cell-cell junction maintenance and adhesive properties) and concurrent acquisition of mesenchymal effector functions (such as migration, ECM remodeling, collagen deposition). This phenotypic conversion recapitulates core features of endothelial-to-mesenchymal transition (EndMT) 10, 35, suggesting its potential involvement in AAA pathobiology and highlighting the need to further investigate this process in AAA progression.

### EndMT is identified in abdominal aorta with aneurysm

**Figure 4:**
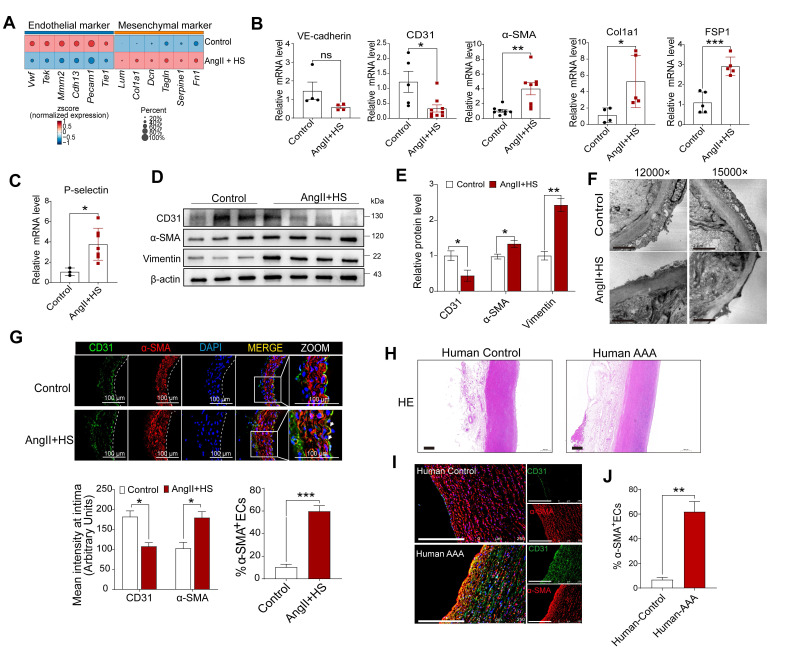
** Endothelial-to-mesenchymal transition (EndMT) occurs in both mouse and human aortic aneurysm.** (A) Dot plots showing the expression of representative endothelial and mesenchymal cell marker genes in the two groups of mice. (B-C) qRT-PCR analysis of CD31, VE-cadherin, α-SMA, FSP1, Col1a1, P-selectin in the aortic intima (n = 3-9). (D-E) Western blotting analysis of CD31, α-SMA and vimentin in isolated aortic ECs from both groups (n = 3-4). (F) Transmission electron microscopy images of abdominal aortas. Scale bar = 2 μm. (G) Immunofluorescence co-staining of CD31 (red) and α-SMA (green) in mouse aortic intima, with DAPI (blue) nuclear counterstain. Scale bar = 100 μm. Arrows indicate CD31^+^ ECs expressing α-SMA. Dashed line indicates the endothelial layer (n = 3). (H) Representative images of hematoxylin and eosin (H&E) staining of human AAA and control aortas. (I) Co-immunofluorescence staining of CD31 and α-SMA in human AAA and control samples. The nuclei were stained blue with DAPI. Scale bar = 250 μm. (J) Quantification of CD31^+^ ECs expressing α-SMA in the aortic intima region (n = 3). *P < 0.05, **P < 0.01, ***P < 0.001 by multiple t-test or unpaired student's t-test (B, C, E, G, J).

Accumulating evidence underscores the fundamental role of EndMT in cardiovascular diseases 12-14. Nevertheless, the mechanistic understanding of EndMT in aortic aneurysm progression remains limited. To bridge this knowledge gap, we systematically evaluated EndMT-related gene expression profiles in VECs across experimental groups. Notably, downregulation of endothelial markers (e.g., *Tie1, Pecam1, Cdh13, Tek,* and* Vwf*) and upregulation of mesenchymal markers (e.g., *Fn1, Acta2, Tagln, Lum, Dcn,* and* Col1a1*) were detected in VECs of AngII+HS-induced AAA mice (Figure 4A). This molecular pattern was further validated in CaCl₂-induced AAA model, showing analogous suppression of endothelial markers (e.g., *Tie1*,* Pecam1*,* Vwf*, and *Cldn5*) and induction of mesenchymal markers (e.g., *Acta2*,* Fn1*,* Fbn1*, and* Dcn*) (Figure S7C). We further confirmed our findings through analysis of existing transcriptomic datasets. Bulk RNA-seq data from deoxycorticosterone acetate (DOCA) and salt-induced mice and microarray data from AngII-treated *ApoE*^-/-^ mice both showed downregulation of endothelial markers and upregulation of mesenchymal markers (Figure S8A-B). Similar expression patterns for EndMT-related genes were observed in human AAA samples (classified by aortic diameter: > 55mm as large AAA, ≤ 55mm as small AAA) through microarray analysis (Figure S8C-D).

To experimentally validate our bioinformatics analysis, we performed a series of biological validations. Aortic intima RNA was isolated for qRT-PCR analysis as described previously 26, 27. As anticipated, endothelial markers (Tie2, VWF, CD31) were highly enriched in the intima and scarce in the media and adventitia, whereas smMHC expression showed the inverse pattern (Figure S9), confirming that the isolated RNA predominantly originated from aortic intima. Quantitative PCR revealed a marked reduction in CD31 and VE-cadherin, accompanied by robust induction of α-SMA, Col1a1, Fsp1 and P-selectin in the aortic intima of AngII+HS-treated mice (Figure 4B-C). Western blotting of isolated ECs from these aortas showed downregulation of CD31 concomitant with upregulation of α-SMA and vimentin relative to controls (Figure 4D-E). Transmission electron microscopy showed that AngII+HS compromised endothelial structure and integrity, as evidenced by irregular cell shape, thinning of the endothelial cell layer, and loss of intercellular junctions (Figure 4F). Immunofluorescence staining of abdominal aortic sections demonstrated reduced CD31 fluorescence intensity paired with increased α-SMA signal in the aortic intima of AngII+HS-induced mice (Figure 4G). Importantly, the fraction of CD31^+^ ECs co-expressing α-SMA increased significantly following AngII+HS treatment (Figure 4G). These findings were corroborated in human AAA samples (Figure 4H-J, Figure S10). Additionally, *en face* immunofluorescence staining tracked the spatiotemporal expression patterns of endothelial marker CD31 and mesenchymal marker FN1 in aortic ECs at 7, 14, and 28 days post-AngII+HS treatment. Time-course analysis revealed progressive reduction of CD31 expression concurrent with gradual increase of FN1 expression in the abdominal aortic endothelium (Figure S11). Further colocalization staining of CD31 and α-SMA identified EndMT-like ECs in AAA mice as early as 1-2 weeks after DOCA plus salt treatment (Figure S12). Our observations support EndMT as a universal process in AAA, with evidence suggesting its potential role as an early event in AAA.

### Sox18 downregulation is critical for promoting EndMT

**Figure 5:**
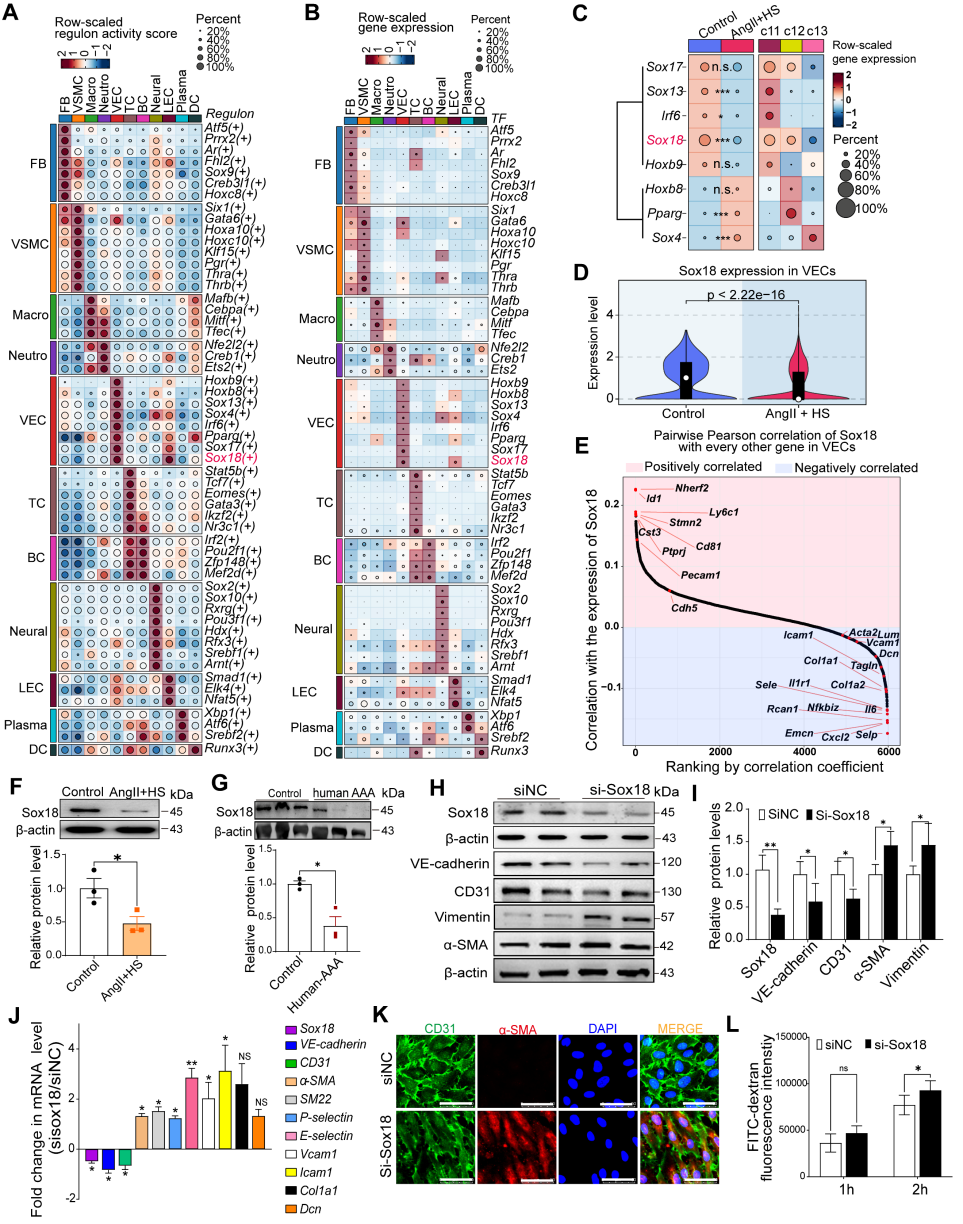
** Sox18 silencing promotes endothelial-to-mesenchymal transition (EndMT).** (A) Dot plot highlighting cell type-specific regulons with relatively high activities in each cell type. (B) Dot plot displaying the expression levels of master transcription factors driving cell type-specific regulons. (C) Dot plot displaying average expression of the master transcription factors for the VEC-specific regulons across groups and VEC clusters (Wilcoxon rank-sum test; *Padj < 0.05, ***Padj < 0.001; n.s., not significant). (D) Violin plots revealed significant Sox18 downregulation in AngⅡ+HS group vs. control group. (E) Pearson correlation analysis revealed positive correlations between the expression of Sox18 and endothelial markers (e.g., *Pecam1 and Cdh5*), and negative correlations with mesenchymal markers (e.g., *Col1a1*, *Tagln*, and *Selp*). (F) Sox18 protein expression in aortic ECs from AngⅡ+HS-treated mice and control mice (n = 3). (G) Decreased Sox18 expression in human AAA (n = 3). (H) Representative protein bands for Sox18, VE-cadherin, CD31, α-SMA and Vimentin in siNC and siSox18 groups. (I) Quantitative analysis of western blot results (n = 4). (J) qRT-PCR analysis showing that Sox18 silencing leads to the reduction of *VE-cadherin* and* CD31*, and the upregulation of *α-SMA*, *SM22α*, *P-selectin*, *E-selectin*, *Vcam1*, *Icam1*, *Col1a1*, *Dcn* (n = 3). (K) Immunofluorescence of CD31 (red) and α-SMA (green) in Sox18-silenced human aortic ECs. The nuclei were stained with DAPI. Scale bar = 50 μm. (L) Transwell permeability assay showing increased FITC-dextran leakage across HAEC monolayers after Sox18 silencing. n = 6. ns, not significant. *P < 0.05, **P < 0.01 by multiple t-test or unpaired student's t-test (F, G, I, J), two-way ANOVA with Tukey's multiple comparisons test (L).

To identify key transcription factors (TFs) governing EC phenotype and EndMT in AAA, we performed SCENIC analysis to identify cell type-specific regulons and transcription factors. Notably, Sox4, Sox13, Sox17 and Sox18 were EC-specific, whereas Gata6 and Sox9 were fibroblast- and VSMC-specific (Figure 5A-B). Moreover, differential expression analysis of the endothelial-specific TFs demonstrated significant alterations in Sox4, Sox13, Irf6, Pparg and Sox18 expression in AngII+HS-treated mice compared with controls, with Sox18 exhibiting the most pronounced difference in expression (Figure 5C). According to our scRNA-seq data and data from The Human Protein Atlas, Sox18 showed the highest level of expression in vascular ECs compared to immune cells, including macrophages (Figure S13A-C). Western blotting analysis confirmed higher Sox18 protein levels in ECs than in macrophages (Figure S13D). Strikingly, Sox18 was significantly downregulated and showed the greatest expression change in ECs of AngII+HS-induced mice (Figure 5C-D). Furthermore, Sox18 levels correlated positively with endothelial marker genes (e.g., *Cdh5* and *Pecam1*) and negatively with mesenchymal marker genes (e.g., *Acta2*, *Tagln*, *Fn1*, and *Col1a2*) (Figure 5E). Bioinformatics analysis revealed that Sox18 downregulation consistently occurred across multiple AAA models, including CaCl_2_-induced AAA mice (Figure S6D), DOCA+HS-treated aortic tissues (Figure S8A), AngII-treated *ApoE*^-/-^ mice (Figure S8B), and human AAA samples (Figure S8C-D), demonstrating conservation across experimental models and human disease. Additionally, we validated the downregulation of Sox18 in aortic ECs of AAA mice induced by AngII+HS (Figure 5F), and in human AAA samples (Figure 5G). Based on these findings, we propose that Sox18 downregulation may initiate EndMT in AAA.

To further validate the above hypothesis, we synthesized a specific Sox18 siRNA. Transfection of HAECs with Sox18 siRNA effectively suppressed Sox18 mRNA and protein levels (Figure 5H-J), accompanied by a significant decrease of endothelial cell markers (VE-cadherin and CD31) and an increase of mesenchymal cell markers (α-SMA, SM22α, P-selectin, E-selectin, VCAM1, ICAM1, Col1a1, Dcn and Vimentin) (Figure 5H-J). Additionally, Sox18 silencing induced cell morphology elongation and promoted co-expression of CD31 and α-SMA in ECs (Figure 5K). Transwell permeability assays demonstrated that endothelial permeability was markedly increased after Sox18 silencing (Figure 5L). These results demonstrate the indispensable role of Sox18 in maintaining normal EC phenotype.

### Sox18 Silencing promotes EndMT through PI3K/Akt signaling pathway

**Figure 6:**
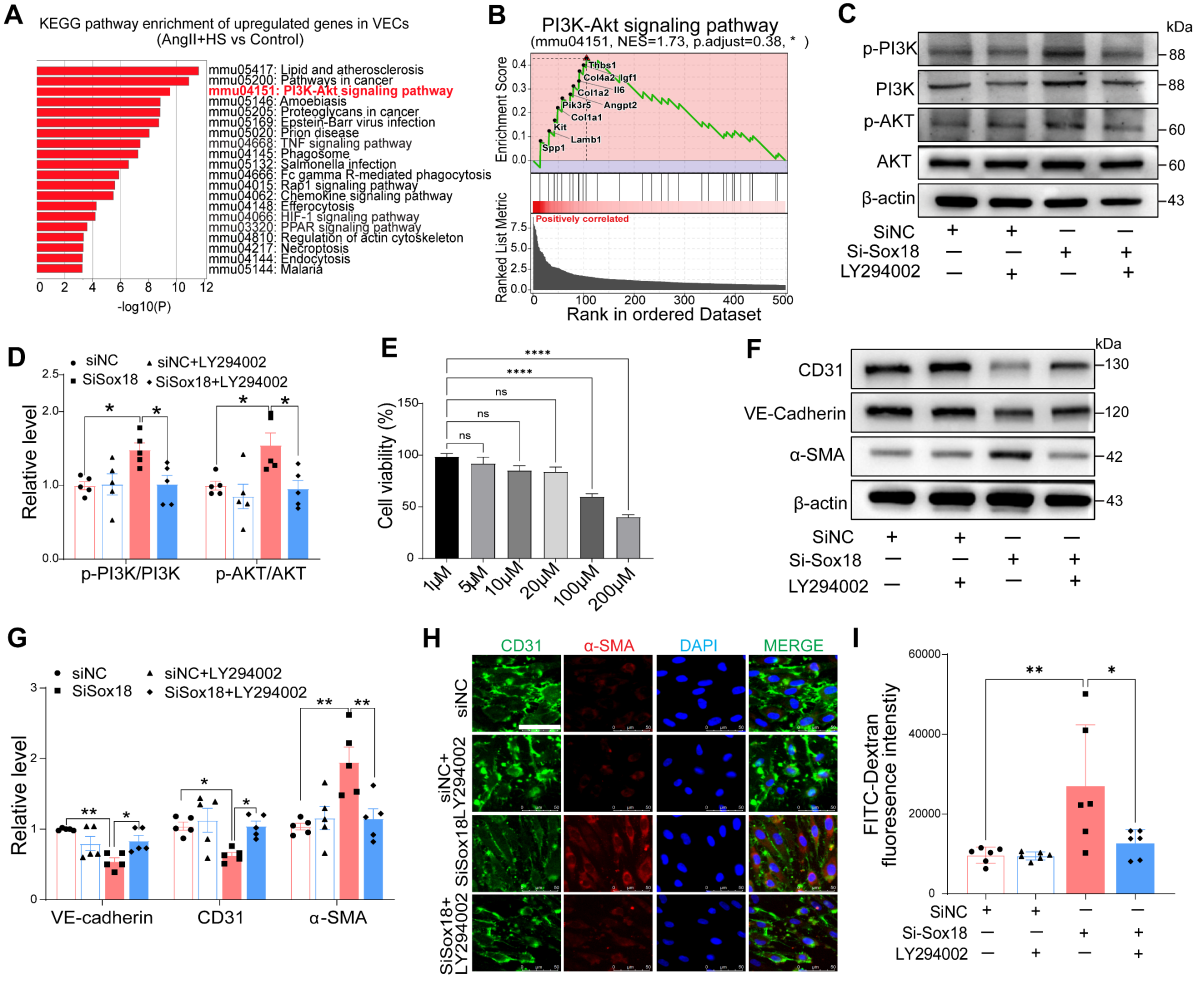
** Sox18 silencing promotes endothelial-to-mesenchymal transition (EndMT) by activating the PI3K/Akt signaling pathway.** (A) KEGG enrichment analysis of the upregulated genes in ECs of AngⅡ+HS group vs. Control group, displaying the top 20 enriched signaling pathways. (B) Gene set enrichment analysis (GSEA) supported the activation of PI3K/Akt signaling pathway in the AngⅡ+HS group. NES, normalized enrichment score. (C-D) Protein levels and quantification of p-PI3K (phosphorylated-PI3K), PI3K (phosphoinositide 3-kinase), p-AKT (phosphorylated-AKT) and AKT (protein kinase B). n = 5. (E) CCK-8 assay to measure EC viability after treatment with different concentrations of LY294002. (F-G) Protein bands and quantification of VE-cadherin, CD31, α-SMA and vimentin (n = 5). (H) Immunofluorescence of CD31 (green) and α-SMA (red) in ECs under different treatments. The nuclei were stained with DAPI. Scale bar = 50 μm. (I) Transwell permeability assay to detect the leak of FITC-dextran across HAEC monolayers. n = 6. ns, not significant. *P < 0.05, **P < 0.01, ****P < 0.0001 by one-way ANOVA with Tukey's multiple comparisons test (D, E, G, I).

Our findings revealed a significant decrease in Sox18 expression within ECs-derived mesenchymal populations. To elucidate how Sox18 downregulation promotes EndMT, we conducted KEGG enrichment analysis on the upregulated genes in ECs from the AngII+HS group compared with the control group (Table S13). The top 20 enriched pathways were shown in Figure 6A. The PI3K/Akt signaling pathway was significantly upregulated after AngII+HS induction. GSEA further confirmed PI3K/Akt signaling activation in AngII+HS group (Figure 6B). Notably, as illustrated in Figure 5E, Sox18 expression exhibited a negative correlation with multiple genes of the PI3K/Akt signaling pathway (e.g., *Col1a1*, *Col1a2*), suggesting that Sox18 silencing may promote EndMT through PI3K/Akt pathway activation. Consistent with this hypothesis, Sox18 knockdown significantly increased p-PI3K and p-AKT protein levels (Figure 6C-D). To further assess the functional role of the PI3K/Akt pathway in Sox18-mediated EndMT, we used the PI3K inhibitor LY294002. After determining 20 μM as the optimal non-cytotoxic concentration (Figure 6E), we found that LY294002 effectively inhibited the upregulation of p-PI3K, p-AKT induced by Sox18 silencing (Figure 6C-D). Critically, LY294002 also prevented the downregulation of endothelial markers (CD31, VE-cadherin) and the upregulation of mesenchymal markers (α-SMA) (Figure 6F-G). Immunofluorescence staining further confirmed that LY294002 attenuated EndMT triggered by Sox18 silencing (Figure 6H). Moreover, LY294002 reversed the increased endothelial permeability caused by Sox18 knockdown (Figure 6I). Similar effects were observed with another PI3K inhibitor XL147, which also attenuated Sox18 silencing-induced EndMT by restoring endothelial markers and reducing mesenchymal marker expression (Figure S14). These results demonstrate that Sox18 silencing promotes EndMT by activating PI3K/Akt signaling pathway.

### Overexpression of Sox18 prevents EndMT and AAA in mice induced by AngⅡ+HS

**Figure 7:**
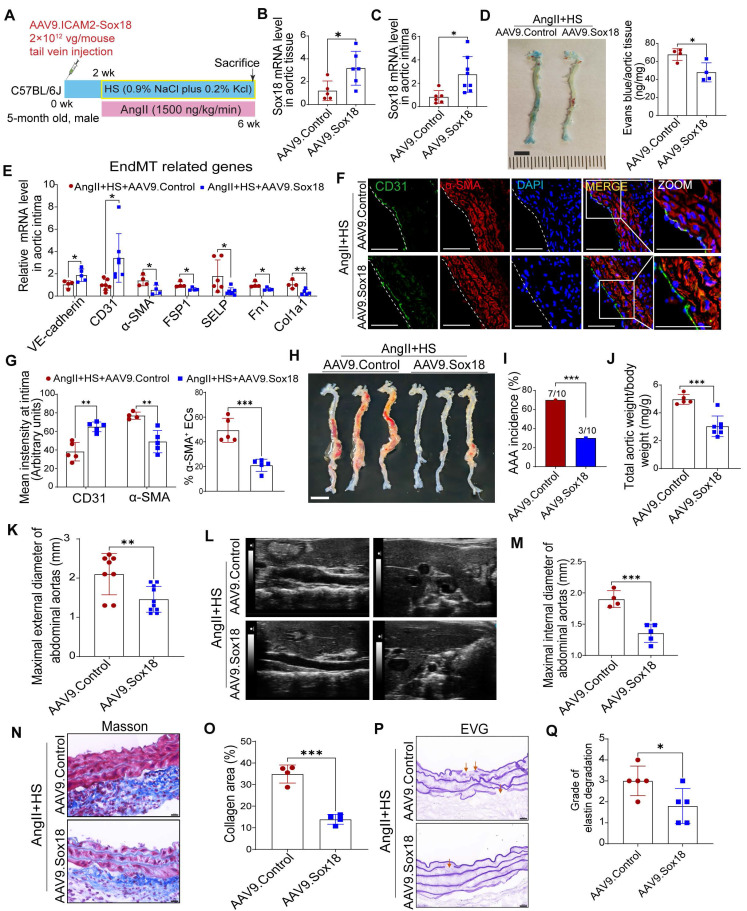
** Sox18 overexpression attenuates abdominal aortic aneurysm (AAA) progression in mice.** (A) Schematic protocol: 5-month-old C57BL/6J mice received intravenous AAV9-Sox18 or AAV9-Control vectors 2 weeks prior to AngⅡ+HS induction. (B) Sox18 mRNA levels in aortic tissues (n = 5- 6). (C) Sox18 mRNA levels in aortic intima (n = 6-8). (D) Evans blue dye staining and quantification in AAV9-Sox18 or AAV9-Control mice 7 days after AngⅡ+HS treatment (n = 4). Scale bar, 4 mm. (E) qRT-PCR analysis of endothelial (CD31, VE-cadherin) and mesenchymal (α-SMA, FSP1, col1a1, p-selectin and Fn1) markers in aortic intima (n = 4-7). (F) Immunofluorescence staining of CD31 and α-SMA in indicated groups. Scale bar = 100 μm. Dashed line indicates endothelial layer. (G) Quantification of aortic intimal CD31 and α-SMA intensity and the percentage of α-SMA^+^ ECs in indicated groups (n = 4-5). (H) Representative macroscopic aortic morphology in two groups of mice. Scale bar, 4 mm. (I) AAA incidence in the two groups of mice. (J) Aortic weight/body weight ratio in indicated groups (n = 5-7). (K) *Ex vivo* external aortic diameter measurements (n = 8-10). (L) Representative ultrasound images of aortas from two groups of mice. (M) Quantification of maximal internal diameter (n = 4-5). (N) Representative Masson staining of mouse abdominal aorta. Scale bar, 20 μm. (O) Quantification of collagen area. n = 4. (P) Representative EVG staining of mouse abdominal aorta. (Q) Quantification of elastin degradation grade. n = 5. *P < 0.05, **P < 0.01, ***P < 0.001 by multiple t-test or unpaired student's t-test (B, C, D, E, G, J, K, M, O, Q), Fisher's exact test (I).

To further investigate the role of Sox18 in EndMT and AAA pathogenesis, we delivered endothelial-specific AAV9-Sox18 (Figure 7A). This approach successfully elevated both mRNA and protein levels of Sox18 in AngII+HS-treated aortic intima (Figure 7B-C, Figure S15A-B). Evans blue permeability assays demonstrated that Sox18 overexpression significantly improved endothelial barrier integrity, as shown by reduced Evans blue dye leakage compared to controls (Figure 7D). qRT-PCR analysis showed that Sox18 overexpression significantly upregulated endothelial marker genes, and downregulated mesenchymal marker genes in the aortic intima (Figure 7E). The decreased percentage of CD31^+^α-SMA^+^ cells further confirmed the inhibitory effect of Sox18 on EndMT (Figure 7F-G). Importantly, endothelial-specific Sox18 overexpression did not affect body weight, plasma lipid levels or blood pressure (Figure S15C-E). However, Sox18 overexpression significantly attenuated AngII+HS-induced AAA formation, as demonstrated by reduced AAA incidence (Figure 7H-I), decreased aortic weight/body weight ratio (Figure 7J), and diminished external and internal diameters of abdominal aortas (Figure 7K-M). Histopathological examination further showed that Sox18 overexpression reduced elastic fibers fragmentation and collagen deposition in AngII+HS-treated mice (Figure 7N-P). These results collectively demonstrate that Sox18 plays a protective role against EndMT and AAA formation *in vivo*.

## Discussion

**Figure 8:**
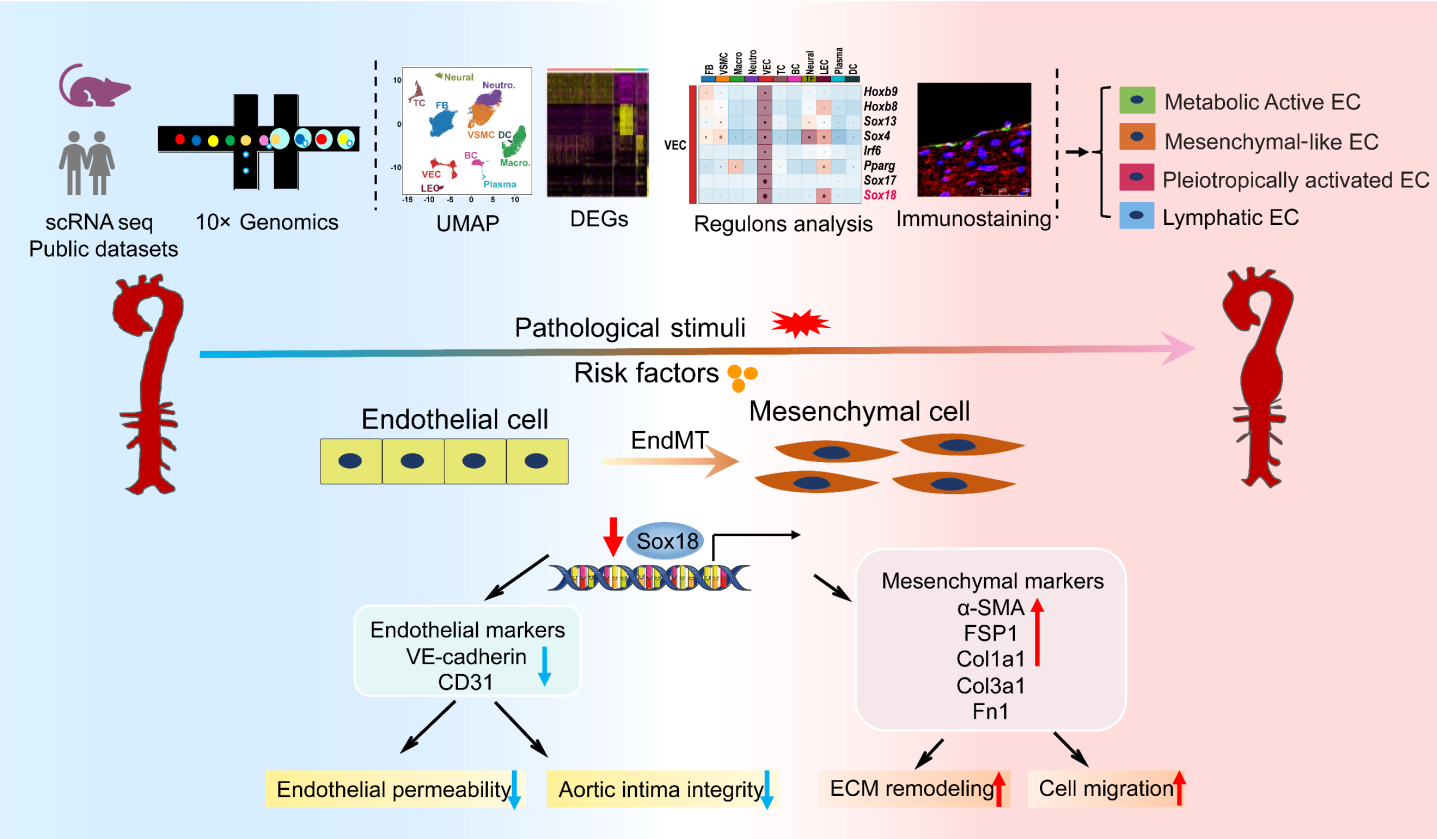
Diagram illustrates that EC heterogeneity and Sox18-mediated EndMT contribute to the pathogenesis of aortic aneurysm.

In this study, we performed scRNA-seq analysis of abdominal aorta from AAA mice induced by AngII+HS and identified 11 distinct cell types. Our analysis further revealed four heterogeneous EC subpopulations in both mouse and human AAA samples, termed *Cd36*^+^metabolically active ECs, *Fn1*^+^ mesenchymal-like ECs, *Lrg1*^+^ pleiotropically activated ECs, and *Mmrn1*^+^ lymphatic ECs. Transcriptional profiling of ECs demonstrated a progressive loss of endothelial characteristics and acquisition of mesenchymal-like gene expression patterns during aortic aneurysm development, providing compelling evidence for EndMT involvement. Through complementary experimental approaches, we validated EndMT occurrence in AAA, and established its role as an early phenotypic alteration of ECs in aneurysm pathogenesis. Mechanistically, we found that Sox18 functioned as a key transcriptional regulator of EndMT by activating the PI3K/Akt signaling pathway. Importantly, Sox18 overexpression significantly attenuated AAA formation and EndMT in mice. These findings elucidate the critical role of EC heterogeneity and Sox18-mediated EndMT in aortic aneurysm development (Figure 8).

Current experimental models for inducing AAA in mice include AngII infusion, elastase perfusion, and calcium chloride or phosphate application 1. While each method replicates certain aspects of human AAA pathology, they all present distinct strengths and limitations 36. The AngII infusion model, though requiring an *ApoE*^-/-^ background in most cases, best recapitulates key features of human AAA. In our study, we successfully established an AAA model in C57BL/6J mice using combined AngII+HS administration and verified its pathological similarity to human AAA. Through scRNA-seq analysis of abdominal aortic aneurysms, we characterized the cellular heterogeneity and compared our findings with previous reports using different AAA models 4, 5, 37. Our results confirmed the universal presence of ECs, VSMCs, fibroblasts, neutrophils, macrophages, B cells, T cells, and DCs in the abdominal aorta from control mice and AAA mice. Aligning with established AAA pathological features, particularly inflammatory cell infiltration and depletion of VSMCs, our scRNA-seq data demonstrated an increase in immune cell populations paralleled by a reduction in VSMCs (Figure 1H). Notably, among all immune cell types analyzed, neutrophils demonstrated both the highest proportional abundance in AAA tissues and the most dramatic changes compared to controls, emphasizing their pivotal role in AAA pathogenesis.

ECs are crucial for maintaining vascular homeostasis, and studies have demonstrated that endothelial dysfunction serves as an early driver of atherosclerosis 38 and aortic aneurysm 8. ECs in normal mouse aorta exhibit well-documented heterogeneity. Kalluri *et al.* identified three EC subpopulations in normal mouse aorta using scRNA-seq 39: lymphatic ECs,* Cd36*^-^/*Vcam1*^+^ canonical ECs, and *Cd36*^+^/*Vcam1*^-^ lipid handling ECs. However, few scRNA-seq studies have focused on EC heterogeneity in aortic aneurysm, with most preferring to characterize monocytes/macrophages, fibroblasts, VSMCs 4, 5, 40. In this study, we identified four EC subpopulations in AAA samples, with a lymphatic EC cluster characterized by high Mmrn1 expression and three other distinct vascular EC subpopulations termed *Cd36*^+^ metabolically active ECs, *Fn1*^+^ mesenchymal-like ECs, *Lrg1*^+^ pleiotropically activated ECs. MMRN1, a carrier protein for platelet factor V/Va, plays a critical role in hemostasis and coagulation 41. This lymphatic EC cluster showed an increase in the cell proportion under AAA conditions (Figure 1H). This increase correlates with intraluminal thrombus formation in abdominal aorta 42 induced by AngII+HS, and suggests lymphatic vessel involvement in AAA, in agreement with previous reports 43-45. We also identified this *Mmrn1*^+^ EC subpopulation in AngII-treated *ApoE*^-/-^ mice, supporting our findings. Our results show that both the lipid-handling EC cluster and mesenchymal-like EC cluster, previously identified in normal mouse aorta 39 and human ascending aorta 46, are consistently present in mouse abdominal aorta and AAA tissues (Figure 2D-E). While total VEC proportion remained similar between groups, the lipid-handling EC cluster (c12) expanded in AAA samples, indicating enhanced lipid metabolic activity. Furthermore, we identified a distinct population of highly activated ECs characterized by Lrg1 expression, which showed upregulated genes involved in inflammatory responses, cell adhesion, coagulation pathways, and migratory processes, suggesting their potential role in AAA pathogenesis.

Our investigation was extended to human AAA samples using published scRNA-seq data (GSE237230) 6. Consistent with our murine findings, we identified four EC subpopulations (EC1-4) in human AAA. Gene expression profiling revealed that EC1 displayed inflammatory properties, EC2 showed mesenchymal-like characteristics with pro-coagulant activity, EC3 exhibited strong cell adhesion properties and endothelial-specific pathway enrichment, and EC4 was associated with high-energy metabolism and active ribosome biogenesis. While human aneurysmal ECs share many features with those in mice, differences exist. Specifically, an EC subpopulation with highly active ribosome function was identified in human AAA but not in mouse AAA. These findings demonstrate that mouse AAA models largely recapitulate human disease characteristics while not fully replicating the complete spectrum of EC heterogeneity. Taken together, EC heterogeneity is consistently present in both mouse and human aortic aneurysm tissues. Although our immunofluorescence experiments validated the existence of four distinct EC subpopulations in the intima of human and mouse aortic aneurysms, we cannot rule out their presence in perivascular adipose tissue (PVAT) or other vascular layers, such as the adventitia. It is well established that ECs exhibit distinct phenotypic and functional differences depending on their anatomical location. While this study primarily focuses on intimal ECs, our findings further underscore the growing recognition of EC heterogeneity in vascular function and aortic aneurysm pathogenesis.

While the contribution of ECs to AAA pathogenesis has historically been understudied, recent work has established the critical role of endothelial barrier integrity in aortic aneurysm development 15, 47. Our scRNA-seq analysis demonstrated that ECs in AAA mice display significantly reduced expression of cell-cell junction genes (e.g., *Vwf*, *Tek*, and *Cdh13*) concomitant with upregulated mesenchymal cell genes (e.g., *Fn1*, *Dcn*, *Lum*, and *Tagln*). These transcriptional changes were accompanied by functional acquisition of migratory capacity, collagen production, and extracellular matrix remodeling activity-hallmark features of EndMT. EndMT is a dynamic process wherein ECs lose their inherent morphology and properties while gaining mesenchymal traits 10. Our biological experiments confirmed EndMT occurrence in human and mouse AAA samples. This observation corroborates the findings of Millar *et al.*, who reported EndMT in elastase-induced murine AAA 48. Notably, our study advances the field through the use of an AngII+HS-induced AAA model that better recapitulates human AAA pathophysiology, including intramural thrombus, dissection, and rupture, compared to elastase models 49. Furthermore, we employed a comprehensive multimodal approach combining scRNA-seq, Western blot analysis of isolated aortic ECs, intimal RNA profiling, immunofluorescence colocalization, and ultrastructural examination by electron microscopy. Taken together, these findings solidify EndMT as a fundamental pathological mechanism in aortic aneurysm formation, offering novel insights into AAA pathogenesis.

Our study identifies Sox18 as a novel regulator in EndMT and AAA. As a member of the SOXF family, Sox18 serves as a master regulator of cellular reprogramming 50. Previous work by Garcia-Flores *et al.* demonstrated the essential role of Sox18 in maintaining endothelial barrier integrity in ventilator-induced lung injury 51. Additionally, Sox18 transduction has been shown to induce endothelial-like features in adipose-derived stem cells 52, and clinical studies have linked a *de novo* Sox18 mutation with aortic dilatation 53. Nevertheless, the relationship between Sox18, EndMT and aortic aneurysm remained unexplored prior to our investigation. Herein, we found significant Sox18 downregulation in the aortic intima of AngII+HS-induced AAA mice, consistent with our scRNA-seq findings. To improve clinical relevance, we extended our analysis to human AAA specimens, where we confirmed reduced Sox18 protein levels. Our *in vitro* and *in vivo* studies further demonstrated the protective role of Sox18 in inhibiting EndMT and mouse AAA. Of course, we cannot exclude the involvement of other transcriptional factors, such as Sox4 25, Sox7 54, and Sox9 55, all of which showed altered expression patterns and merit further investigation.

Accumulating evidence indicates that endothelial barrier dysfunction represents an early event in AAA pathogenesis 15. Given that EndMT compromises endothelial barrier function, our findings provide additional support for its potential involvement in the initial stage of AAA. Although our immunofluorescence analysis identified ECs co-expressing mesenchymal marker in the intimal layer as early as 7 days post-AAA induction, these results do not definitively establish whether EndMT represents an early and persistent process throughout AAA progression. A complete understanding of EndMT dynamics in AAA will necessitate future studies utilizing endothelial lineage tracing techniques or longitudinal single-cell RNA sequencing of abdominal aortic tissues. These approaches would allow comprehensive monitoring of endothelial phenotypic transitions during disease evolution. Despite these limitations, our current findings suggest that therapeutic strategies targeting EndMT or activated ECs may offer promising opportunities for early AAA intervention.

In conclusion, our study reveals four distinct EC subpopulations that undergo differential proportional changes during AAA development, underscoring EC plasticity and emphasizing the critical involvement of inflammation, lipid metabolism, and ECM remodeling in aortic aneurysm pathogenesis. More importantly, we provide compelling evidence that EndMT is a unifying phenomenon in AAA, and we identify Sox18 as a novel therapeutic target for mitigating AAA through modulation of EndMT. These discoveries not only advance our understanding of AAA pathogenesis but also pave the way for developing innovative EC-based therapeutic strategies for aortic aneurysmal diseases.

## Supplementary Material

Supplementary figures and table 1.

Supplementary tables 2-13.

## Abbreviations

AAA: abdominal aortic aneurysm
scRNA-seq: single-cell RNA sequencing
GSEA: gene set enrichment analysis
GO-BP: gene ontology biological processes
ECs: endothelial cells
EndMT: endothelial-to-mesenchymal transition
Sox18: SRY (sex-determining region on the Y chromosome)-box transcription factor 18
ECs: endothelial cells
VSMCs: vascular smooth muscle cells
ECM: extracellular matrix
AngII: angiotensin II
DCs: dendritic Cells
NK: natural killer
DEGs: differentially expressed genes
HAECs: human aortic endothelial cells
FITC: fluorescein isothiocyanate
DOCA: deoxycorticosterone acetate
H&E: hematoxylin and eosin
TG: triglyceride
TCHO: total cholesterol
LDL-C: low-density lipoprotein cholesterol
HDL-C: high-density lipoprotein cholesterol
