# Supplementary material for: Single-cell RNA sequencing reveals endothelial cell heterogeneity and Sox18-mediated EndMT in abdominal aortic aneurysm
Source: Theranostics. 2025 Aug 30;15(18):9486–507. doi: 10.7150/thno.110254 (PMC12486145; doi:10.7150/thno.110254)
Supplement: Supplementary file 1 — Supplementary figures and table 1. [file thnov15p9486s1.pdf]

Supplementary items

Table S1 to S13 (including table legends)

Figure S1 to S15 (including figure legends)

Supplemental tables

Table S1. Patients' information for human AAA samples.

Table S2. Primers used for qRT-PCR.

Table S3. Marker genes for all subclusters.

Table S4. GO-BP enrichment for cell type marker genes.

Table S5. Filtered marker genes for vascular endothelial cells (VECs) subclusters.

Table S6. EC marker genes and differentially expressed genes (DEGs) across EC1-EC4 (scRNA-seq, GSE186865).

Table S7. GO enrichment of DEGs in EC1-EC4 (scRNA-seq, GSE186865).

Table S8. EC marker genes and differentially expressed genes (DEGs) across EC1-EC4 (scRNA-seq, GSE237230).

Table S9. GO enrichment of DEGs in EC1-EC4 (scRNA-seq, GSE237230).

Table S10. Enriched GO terms for upregulated VEC genes (AngII+HS vs. control).

Table S11. Enriched GO terms for downregulated VEC genes (AngII+HS vs. control).

Table S12. GSEA results in VECs of mouse aorta (AngII+HS vs. control).

Table S13. KEGG enrichment for upregulated VEC genes (AngII+HS vs. control).

Table S1. Patients' information for AAA samples

Patient number	Age	Gender	Infrarenal AAA size (cm)
1	74	Male	5.63
2	64	Male	5.42
3	72	Male	5.52

Human abdominal aortic samples were obtained from patients with abdominal aortic aneurysm (AAA) who underwent open aortic repair. The control samples were resected from the adjacent normal aortic segments from the same patient. AAA size was determined by ultrasound scanning.

Supplemental figures

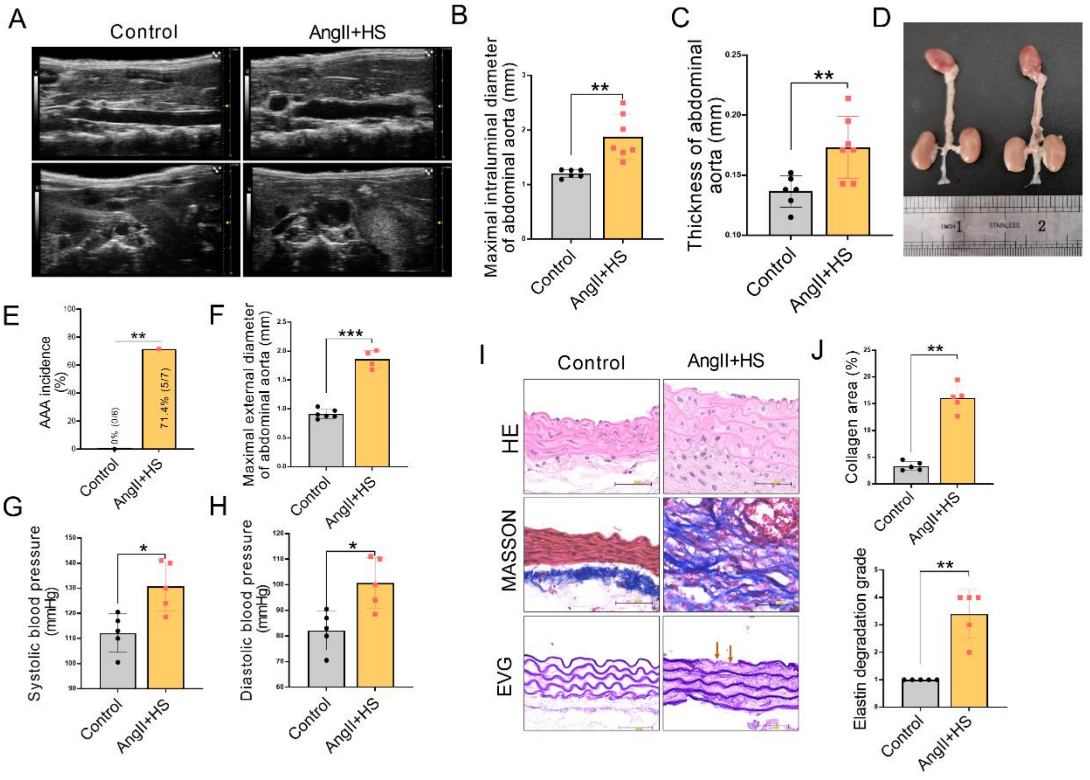

Figure S1. AngII+HS induced abdominal aortic aneurysm formation in C57BL/6J mice. (A) Representative ultrasound images of aortas from control mice and mice treated with AngII+HS. (B) Quantification of intraluminal diameter of the abdominal aorta based on ultrasound data (n = 6-7). (C) Quantification of the abdominal aorta thickness based on ultrasound data (n = 6-7). (D) Representative photographs of aortas from the two groups. (E) Incidence of abdominal aortic aneurysm. (F) Quantification of maximal external diameter of the abdominal aorta (n = 4-6). (G-H) Systolic and diastolic blood pressure (n = 5). (I) Representative photomicrographs of HE, Masson and Elastin staining of the abdominal aorta from control and AngII+HS groups. Scale bar = 50 μm. (J) Quantification of collagen deposition and elastin degradation scores. n = 5, *P<0.05; **P<0.01; ***P<0.001 vs. Control mice by unpaired Student's t-test (B, C, F, G, H), Fisher's exact test (E).

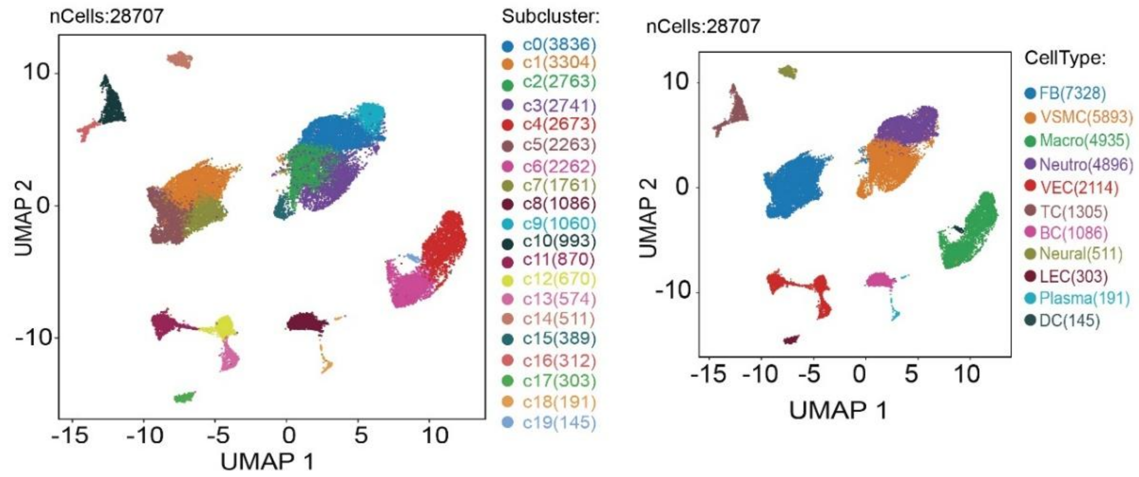

Figure S2. Unsupervised clustering of the combined dataset (comprising 28,707 cells from all samples) identifies 20 cell clusters and 11 cell types.

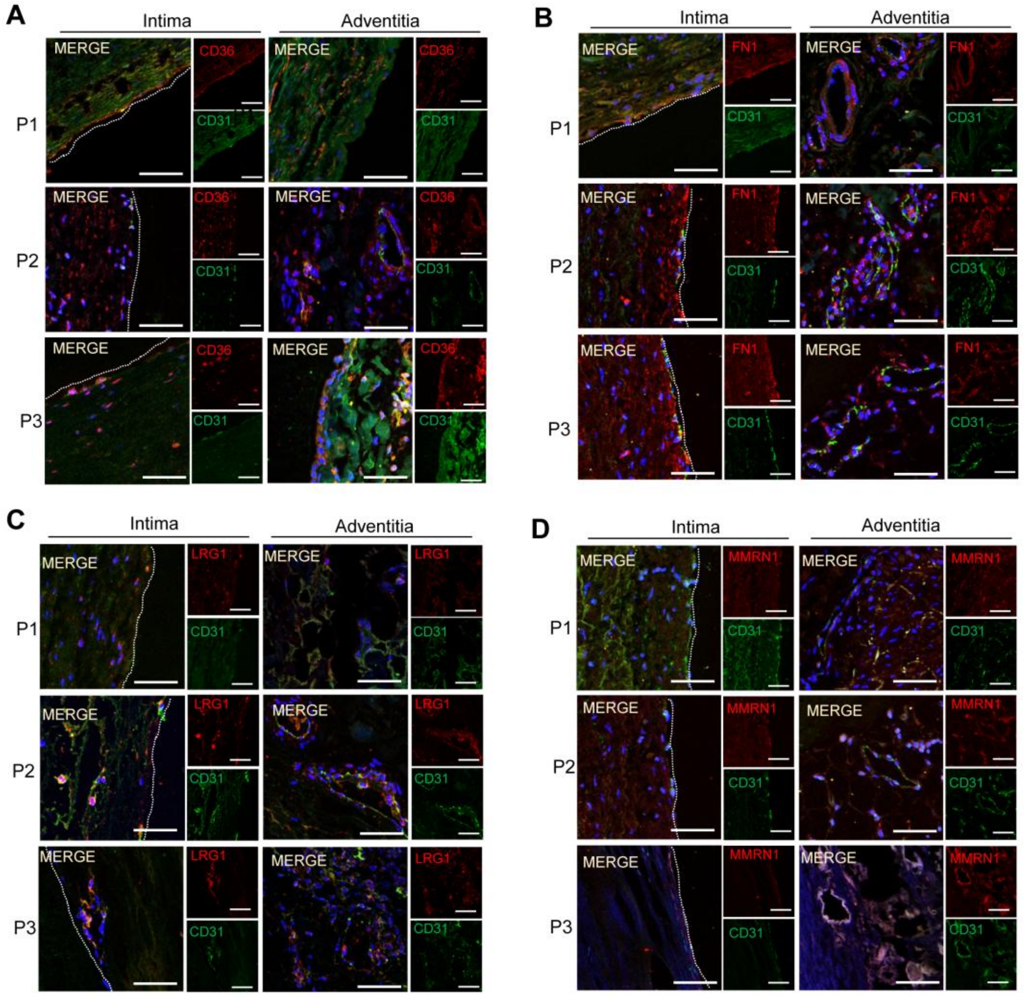

Figure S3. Immunofluorescence staining detected the presence of four EC subpopulations in three human AAA samples. (A): CD31^{+}CD36^{+}, (B): CD31^{+}FN1^{+}, (C): CD31^{+}LRG1^{+}, (D): CD31^{+}MMRN1^{+}. Scale bar = 50 μm. Dashed line indicates the

endothelial layer. P1: Patient 1, P2: Patient 2, P3: Patient 3.

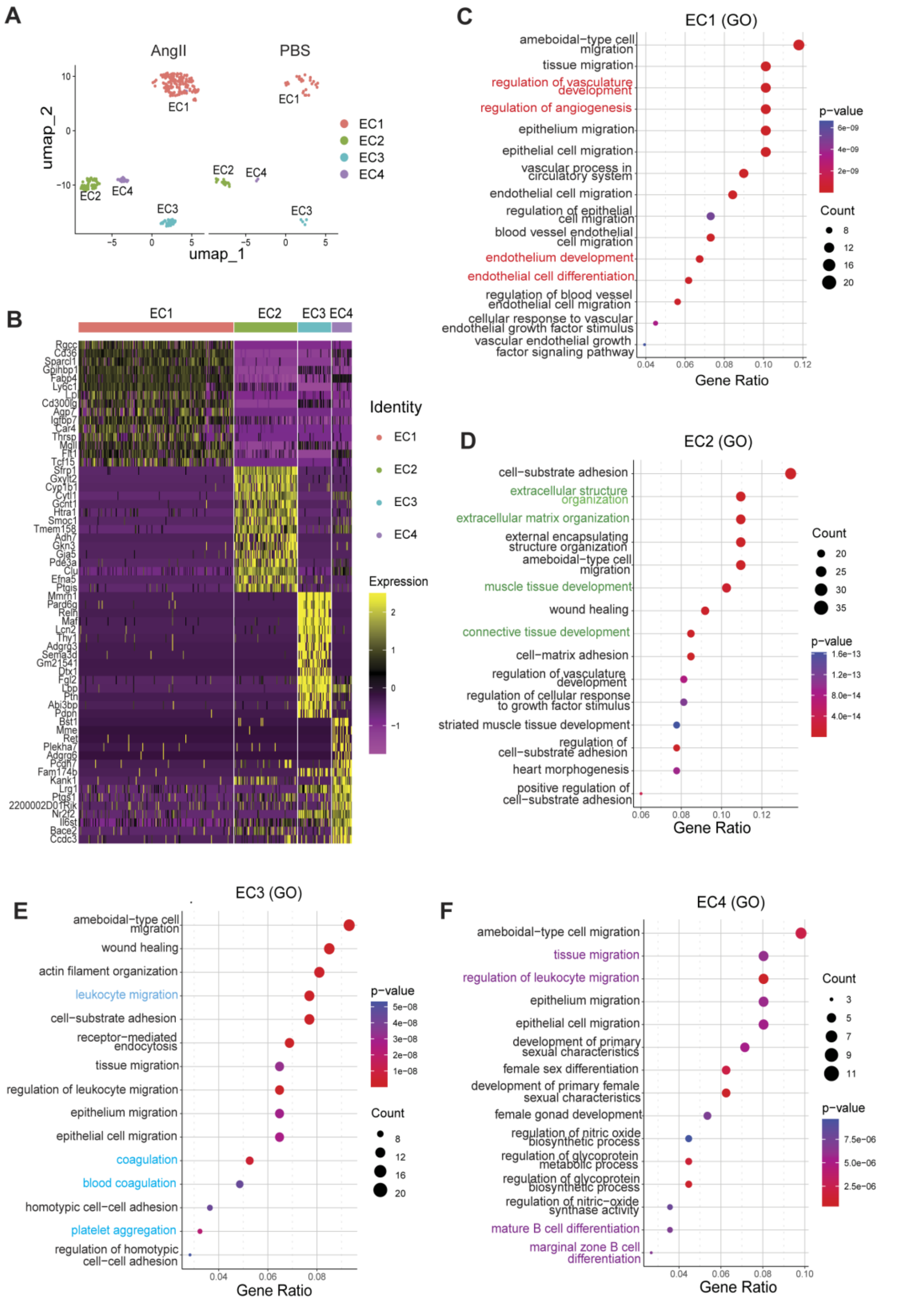

Figure S4. Characterization of EC subpopulations in mouse AAA samples induced by AngII in *ApoE*$^{-/-}$ mice. (A) UMAP plot showing the EC subpopulations in the aorta of *ApoE*$^{-/-}$ mice treated with PBS and AngII. (B) Heatmap showing the top 15 marker genes among the four EC subpopulations. (C-F) GO enrichment analysis of upregulated DEGs in each EC population compared with the other three EC populations.

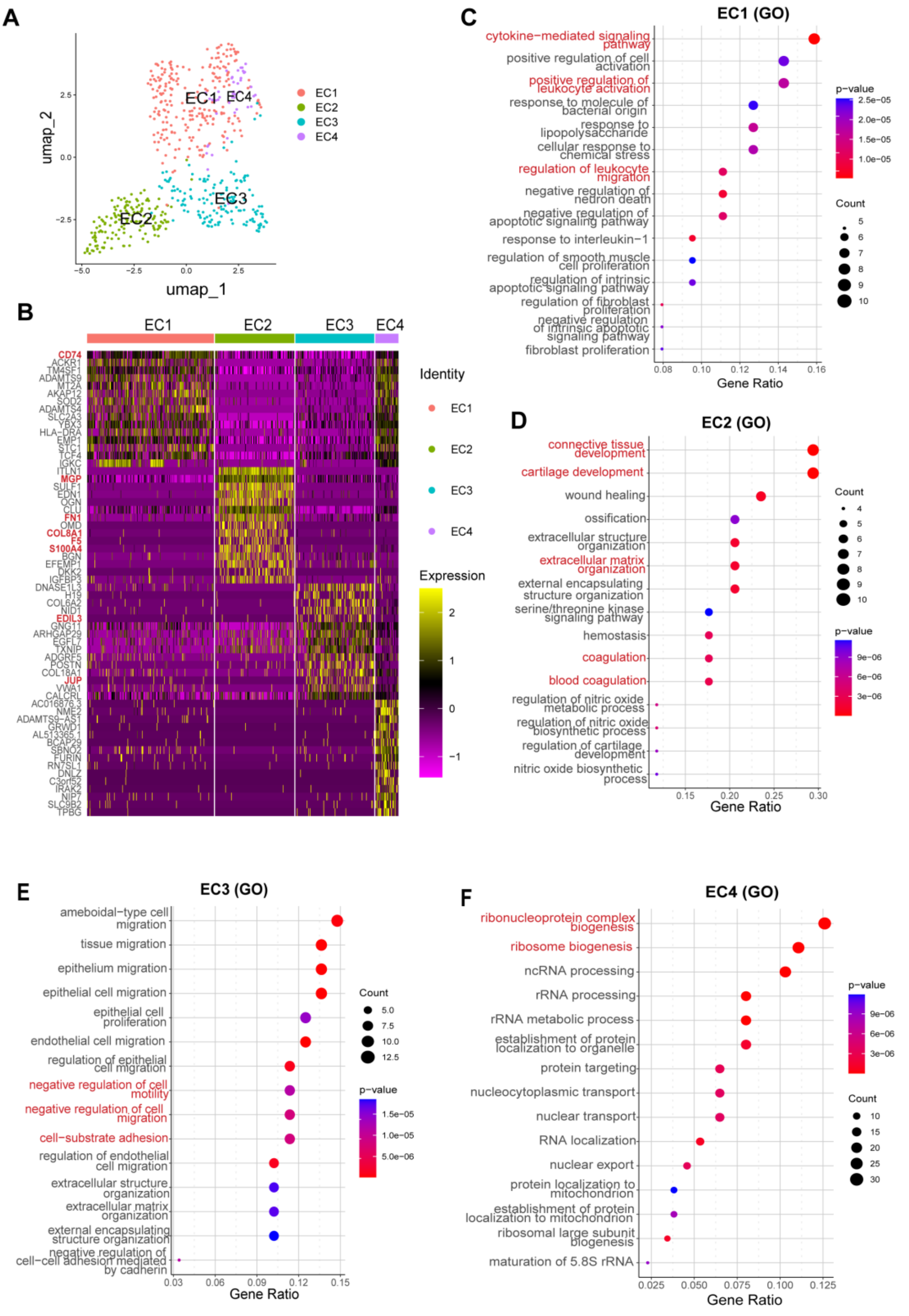

Figure S5. Characterization of EC subpopulations in human AAA samples. (A) UMAP plot showing the EC subpopulations from human aneurysmal aortas. (B) Heatmap showing the top 15 marker genes among the four EC subpopulations. (C-F) GO enrichment analysis of upregulated DEGs in each EC population compared with

the other three EC populations.

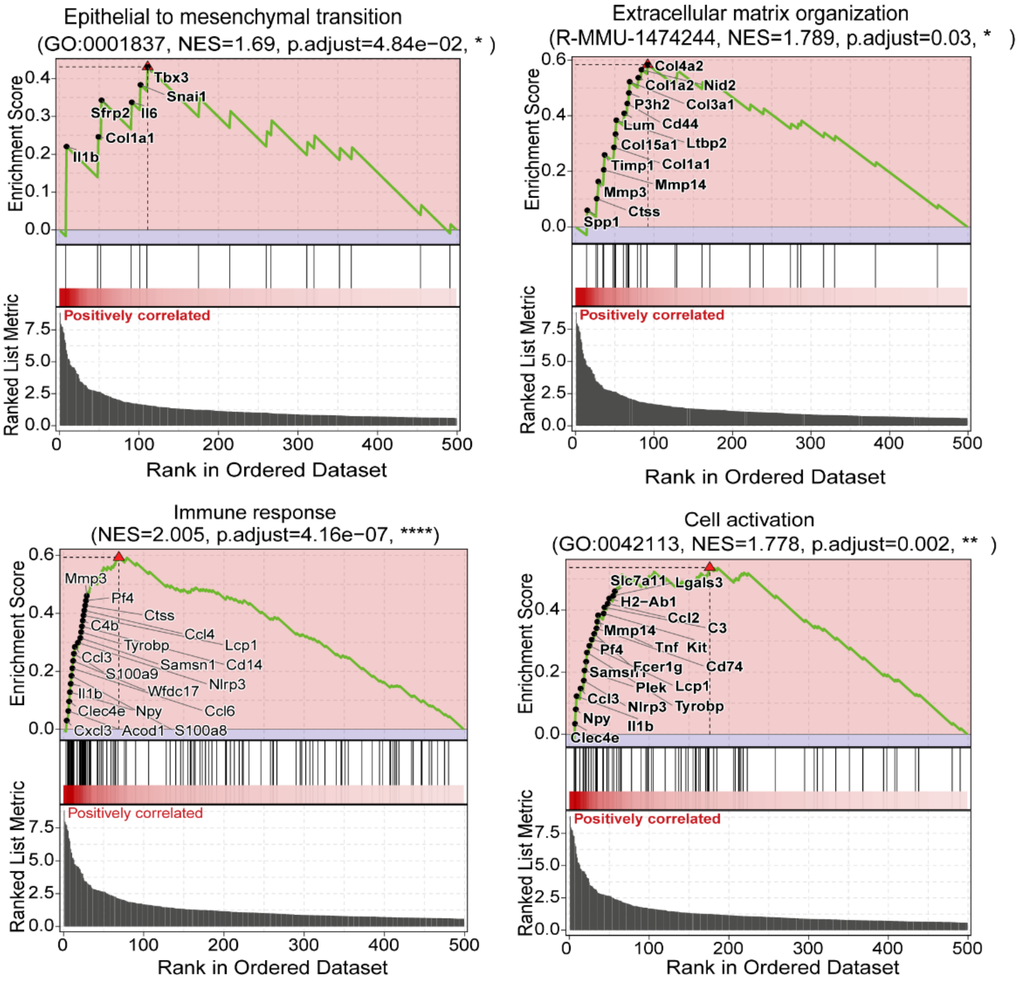

Figure S6. Gene set enrichment analysis (GSEA) showing the activation of epithelial to mesenchymal transition (EndMT), extracellular matrix organization and immune response in ECs of the AngII+HS group.

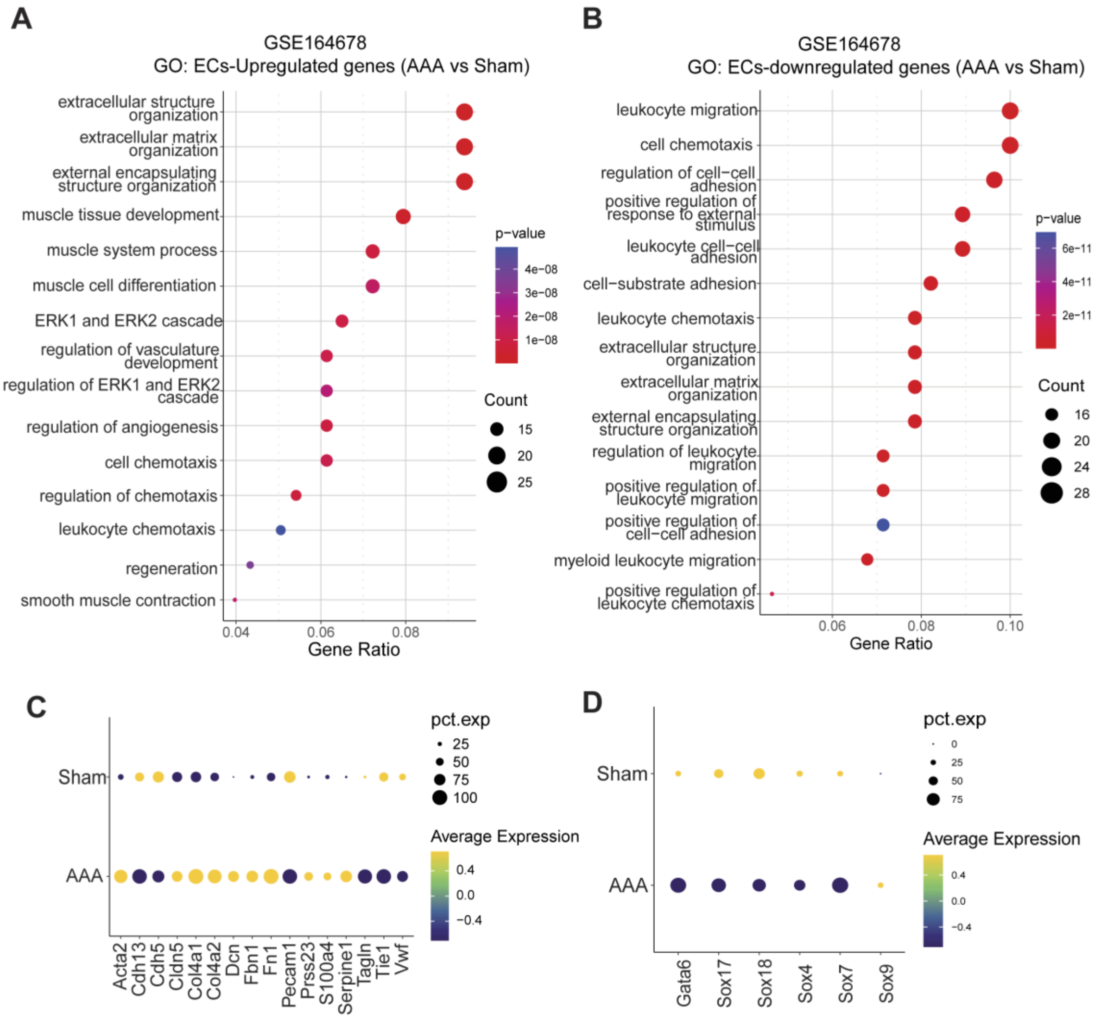

Figure S7. Abdominal aortic ECs show transcriptional alterations in the $CaCl_2$-induced AAA mice. (A) GO enrichment analysis of upregulated genes in AAA vs. Sham. (B) GO enrichment analysis of downregulated genes in AAA vs. Sham. (C) Dot plots showing the expression of endothelial cell marker genes and mesenchymal marker genes. (D) Dot plots showing the expression of several transcriptional factors.

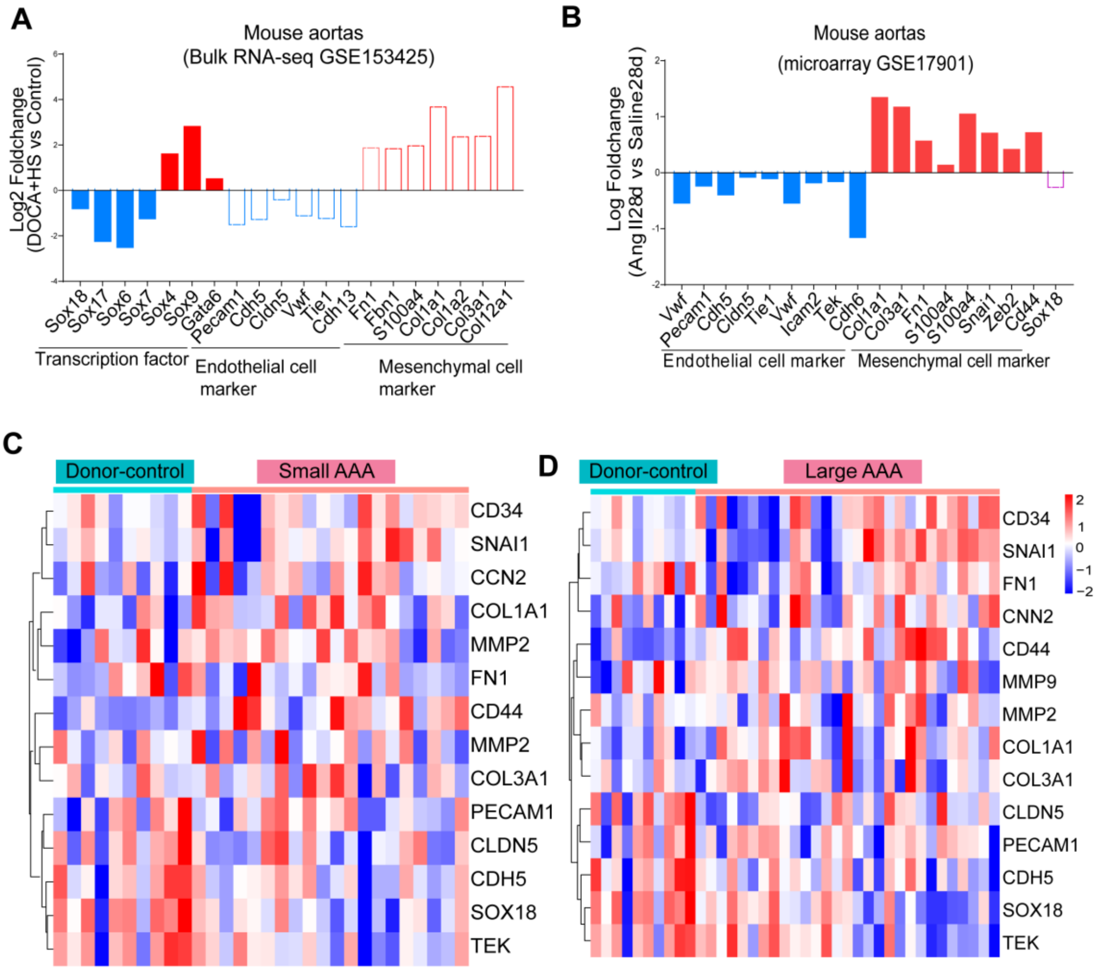

Figure S8. Bioinformatic analysis to detect EndMT-related gene expression in aortic tissues of mouse and human AAA. (A) Bulk RNA-seq analysis showing the expression changes of endothelial cell marker genes, mesenchymal marker genes and several related transcriptional factors in mouse aortic tissues induced by DOCA+HS. (B) Microarray data analysis showing the expression changes of endothelial cell marker genes, mesenchymal marker genes and Sox18 in aortic tissues of *ApoE*$^{-/-}$ mice treated with AngII. (C-D) Heatmap showing the normalized expression levels of endothelial cell marker genes, mesenchymal marker genes and Sox18 in aortic tissues from human AAA samples (Small AAA; abdominal aorta ≤ 55 mm; large AAA, abdominal aorta > 55 mm) by microarray data analysis (GSE57691).

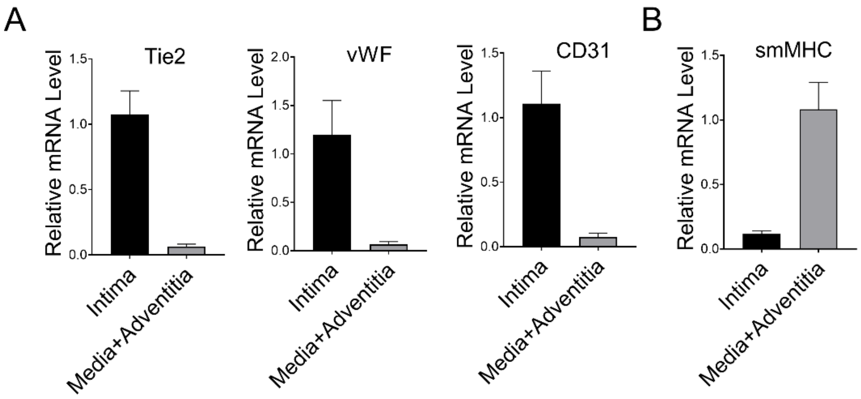

Figure S9. Characterization of extracted RNA from aortic intima. qRT-PCR analysis of (A) endothelial markers Tie2, vWF and CD31, and (B) smooth muscle cell marker smMHC in the aortic intima, media and adventitia. Data show mean ± SEM, n = 5.

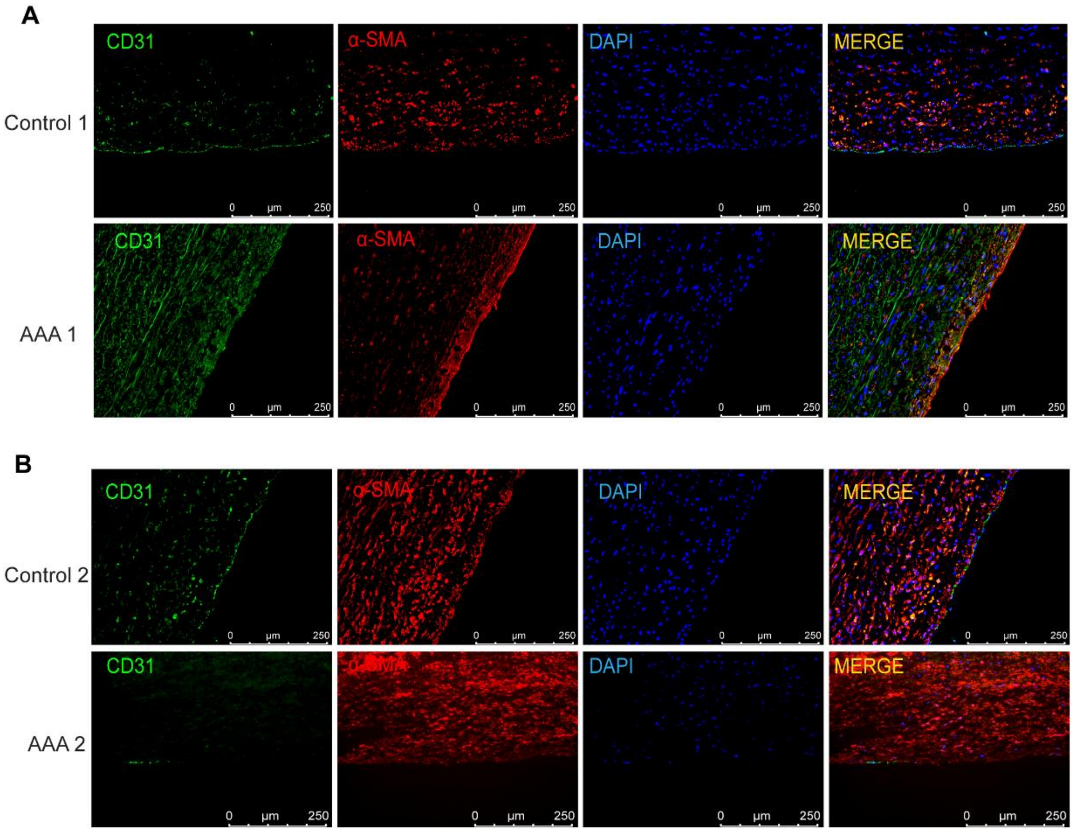

Figure S10. Co-immunofluorescence staining of CD31 and α-SMA in human AAA and control samples. The nuclei were stained blue with DAPI. Scale bar = 250 μm.

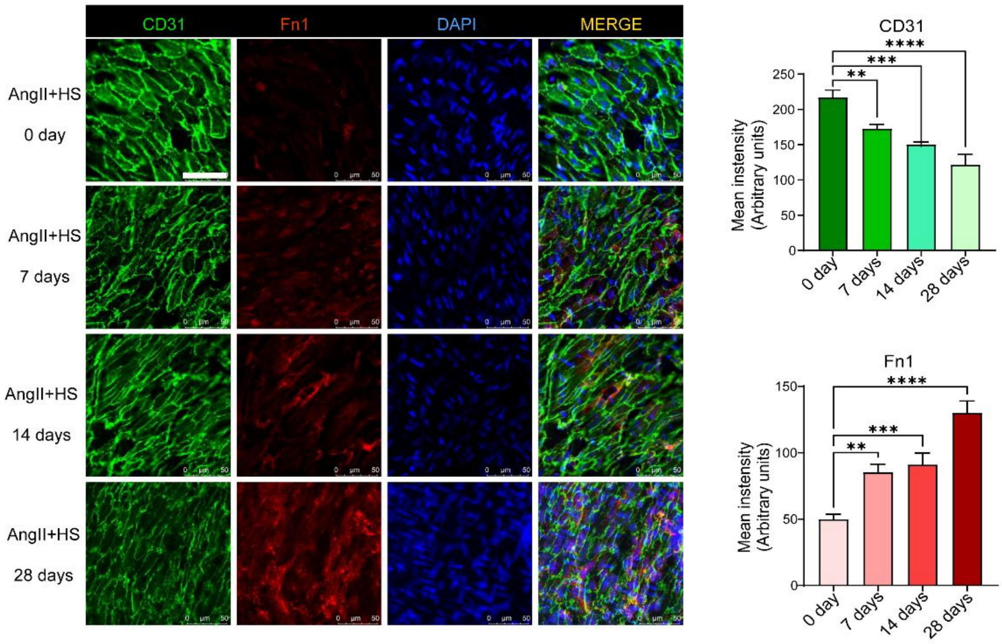

Figure S11. *En face* immunofluorescence staining showing CD31 and Fibronectin (Fn1) expression in the abdominal aortas of mice following AngII+HS treatment. DAPI was used to visualize nuclei. Scale bar, 50 μm. Right graphs: quantification of CD31 and Fn1 fluorescence intensities (n = 3). ***P* <0.01, ****P* <0.001, *****P* <0.0001 by one-way ANOVA with Tukey's multiple comparisons test.

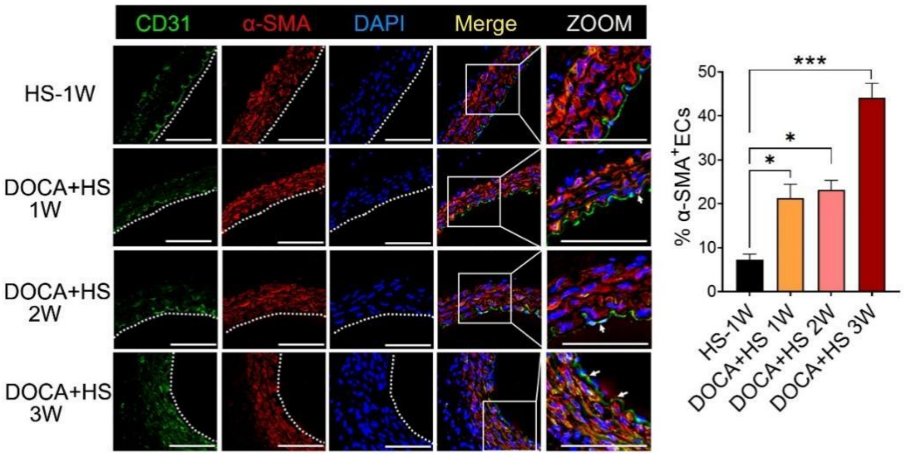

Figure S12. Co-immunofluorescence staining of CD31 and α-SMA in the abdominal aorta of mice induced by DOCA+HS for different times. The nuclei were stained blue with DAPI. Scale bar = 100 μm. 1w, 1 week; 2w, 2 weeks; 3w, 3 weeks. Dashed lines indicate the endothelial layer. Right graph: Percentage of α-SMA^{+} ECs in the aortic intima (n = 3). **P* <0.05, ****P* <0.001 by one-way ANOVA with Tukey's multiple comparisons test.

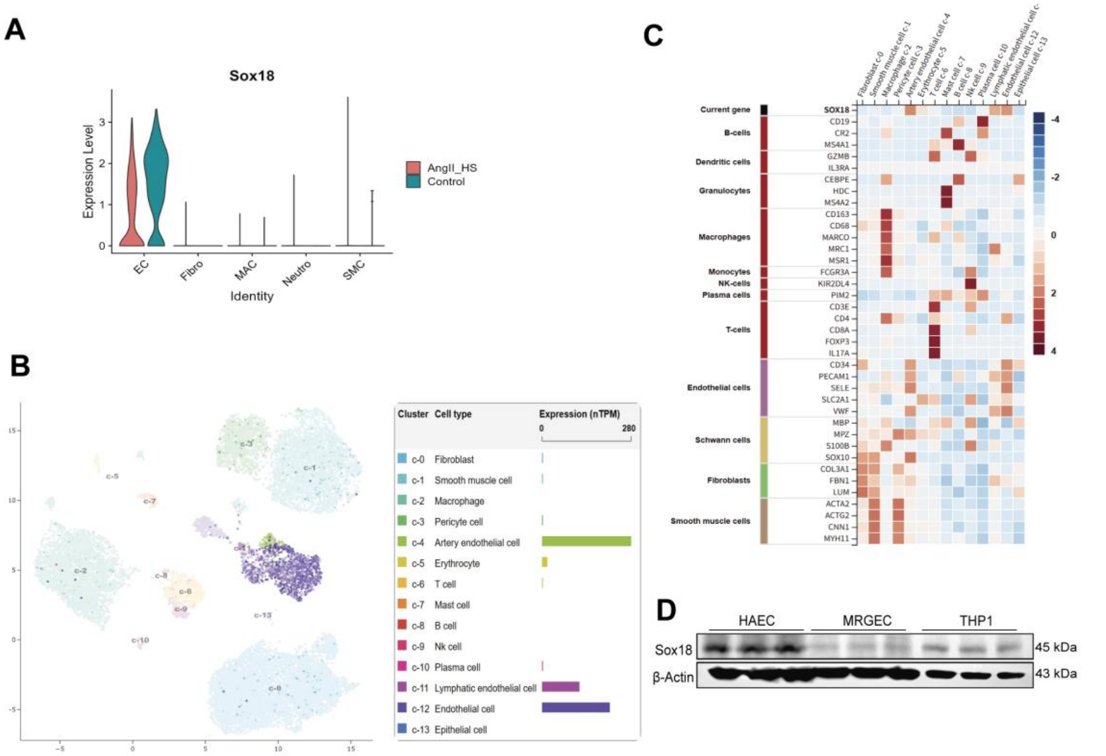

Figure S13. Sox18 expression in different vascular cells. (A) Violin plots showing the expression of Sox18 in different vascular cells from the control and AngII+HS group. (B-C) Sox18 expression in different cell types of vascular tissues from The Human Protein Atlas (https://www.proteinatlas.org/ENSG00000203883-SOX18/single+cell/vascular). (D) Western blotting showing the protein levels of Sox18 in ECs and THP1 cells.

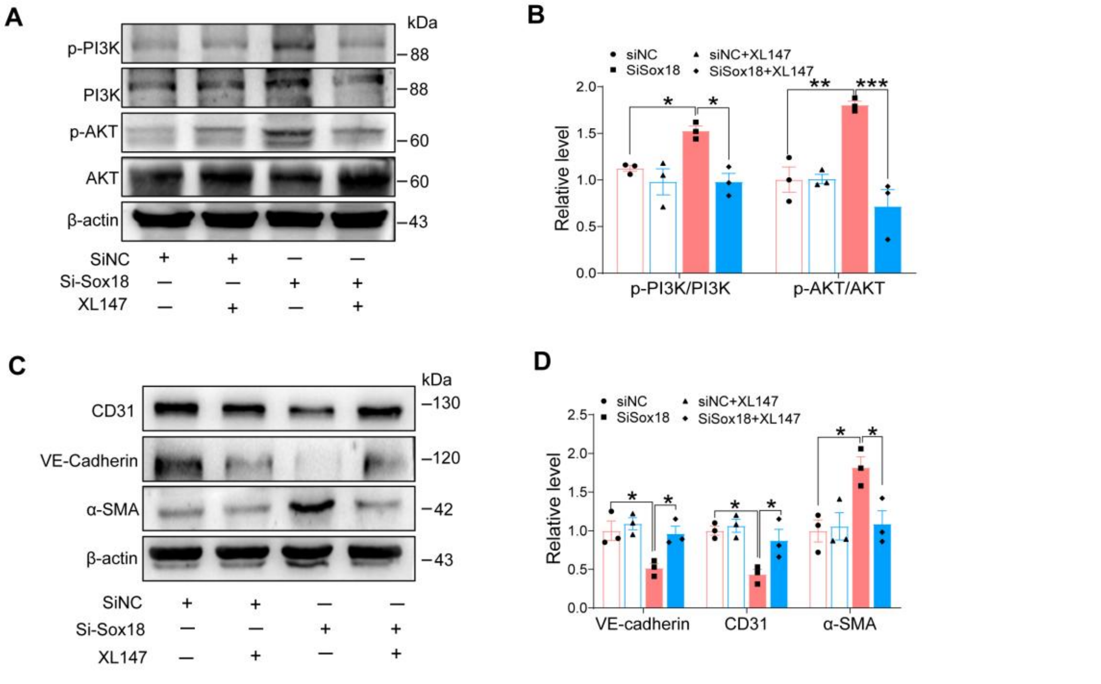

Figure S14. PI3K inhibitor XL147 prevents EndMT induced by Sox18 silencing. (A-B)

Protein levels and quantification of p-PI3K (phosphorylated-PI3K), PI3K (phosphoinositide 3-kinase), p-AKT (phosphorylated-AKT) and AKT (protein kinase B). n = 3. (C-D) Protein bands and quantification of VE-cadherin, CD31 and α-SMA. n = 3. **P* <0.05, ***P* <0.01, ****P* <0.001 by one-way ANOVA with Tukey's multiple comparisons test (B, D).

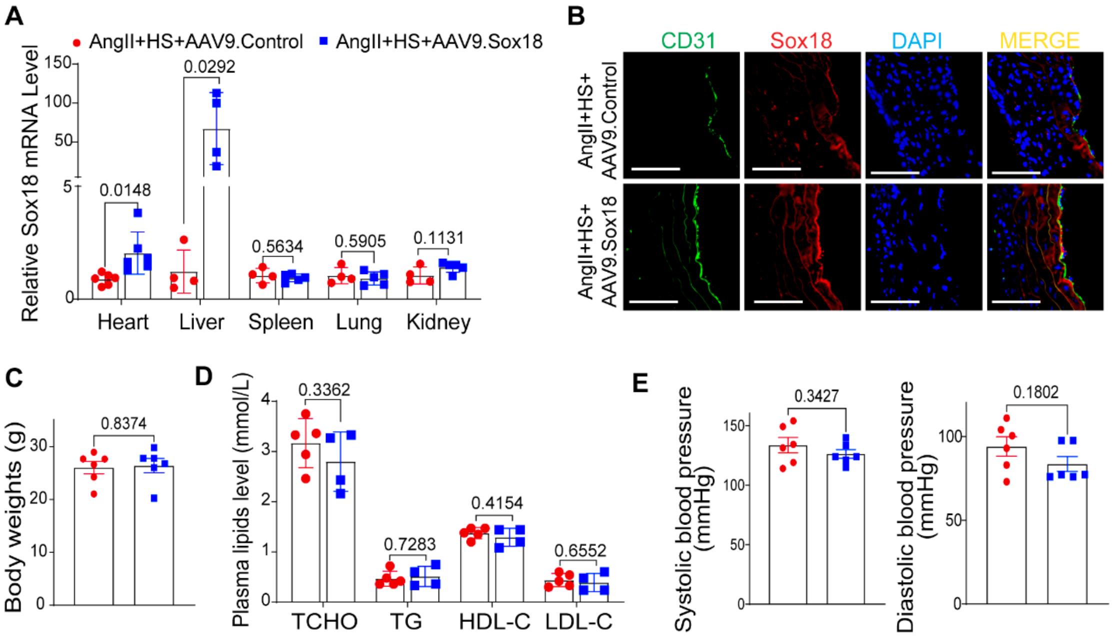

Figure S15. Endothelial-specific Sox18 overexpression does not alter baseline characteristics in AngII+HS-treated mice. (A) Sox18 mRNA levels across tissues (n = 4-6). (B) Co-immunofluorescence staining showing Sox18 expression in the aortic intima of mice. Scale bar = 100 μm. (C) Body weight in two groups of mice (n = 6). (D) Plasma lipids levels in two groups of mice (n = 4-5). TCHO, total cholesterol; TG, triglyceride; HDL-C, High-density lipoprotein cholesterol; LDL-C, Low-density lipoprotein cholesterol. (E) Blood pressure in two groups of mice (n = 6-7). Data are represented as mean ± SEM. Statistical significances were determined by multiple t-tests or unpaired Student's t-test.
